# Supplementary figures and images for: The response of culturally important plants to experimental warming and clipping in Pakistan Himalayas
Source: PLoS One. 2021 May 6;16(5):e0237893. doi: 10.1371/journal.pone.0237893 (PMC8101745; doi:10.1371/journal.pone.0237893)

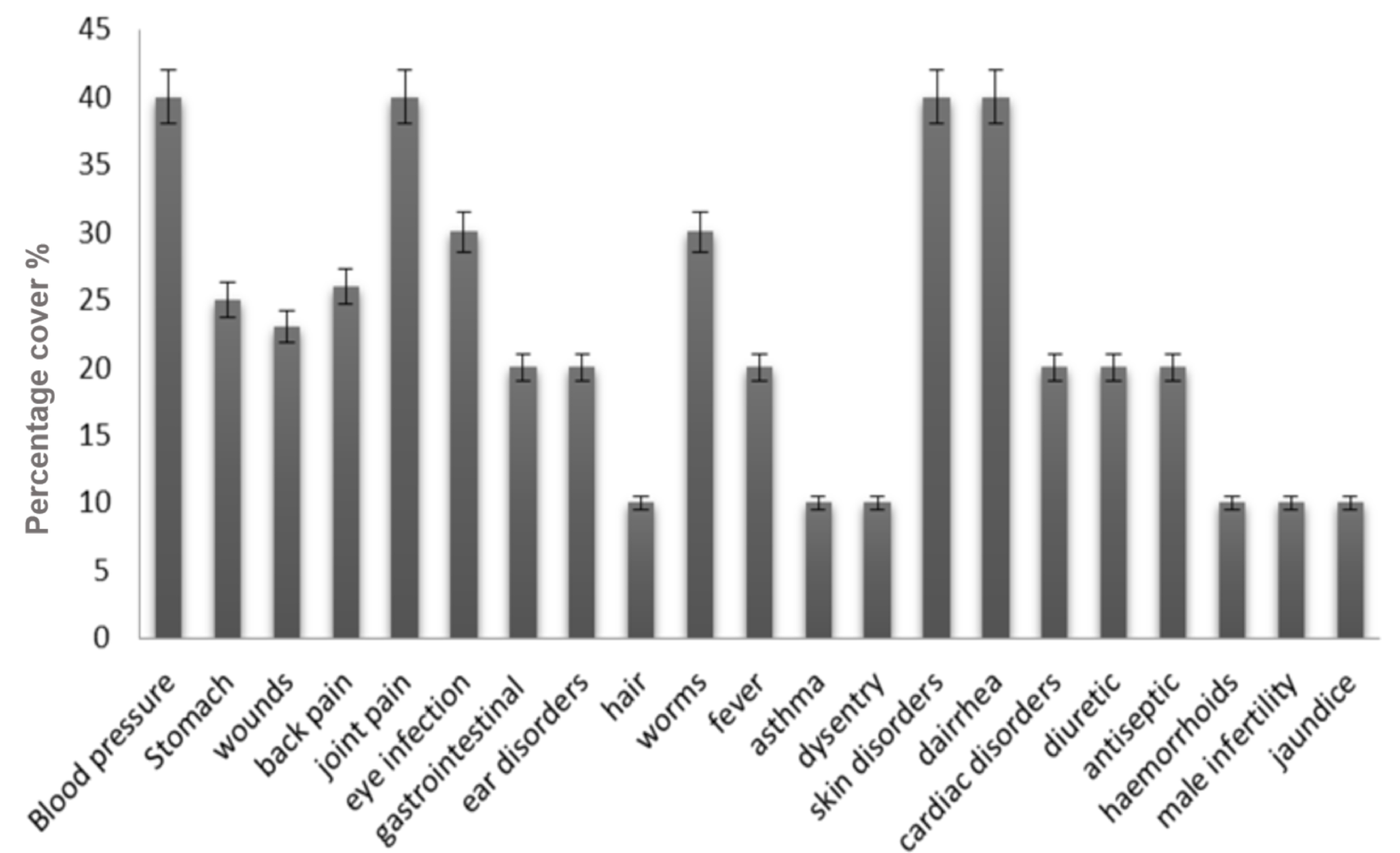

Supplement: S1 Fig — Percent responses of informants for different diseases treated by medicinal plants. (TIFF) [file pone.0237893.s001.tiff]

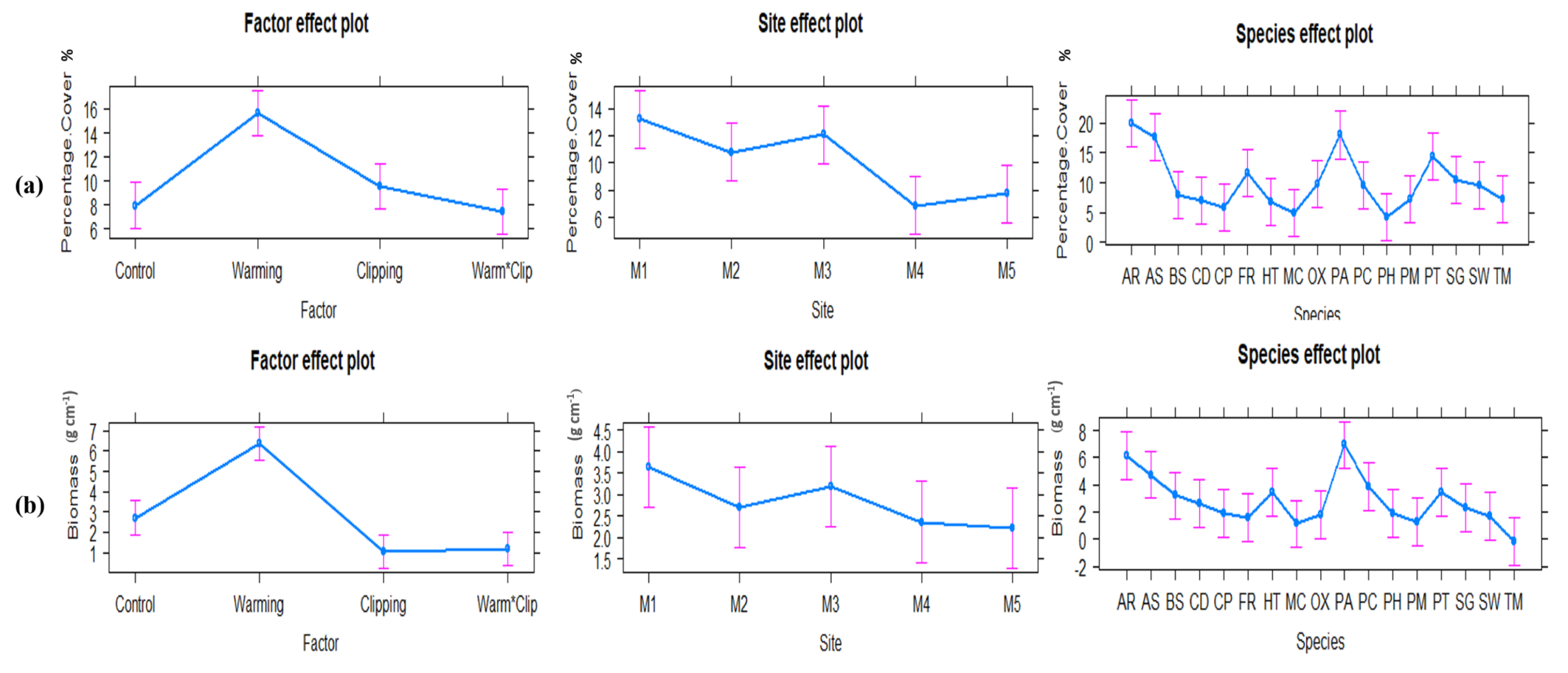

Supplement: S2 Fig — a) the percentage cover of plant species increase in warming treatment and at multiple sites(M1, M2, M3) that is because of high abundance of these species on these sites while among species the response of individual species was not very different from each other but some species responded more positively by increasing their percent cover as AR and AS b) there is a decline in biomass of species in clipping treatment as compared to control but the significant positive effect of warming treatment. The side effect is not very different among all site similarly some species biomass increases while others received a decrease in the biomass. (TIFF) [file pone.0237893.s002.tiff]

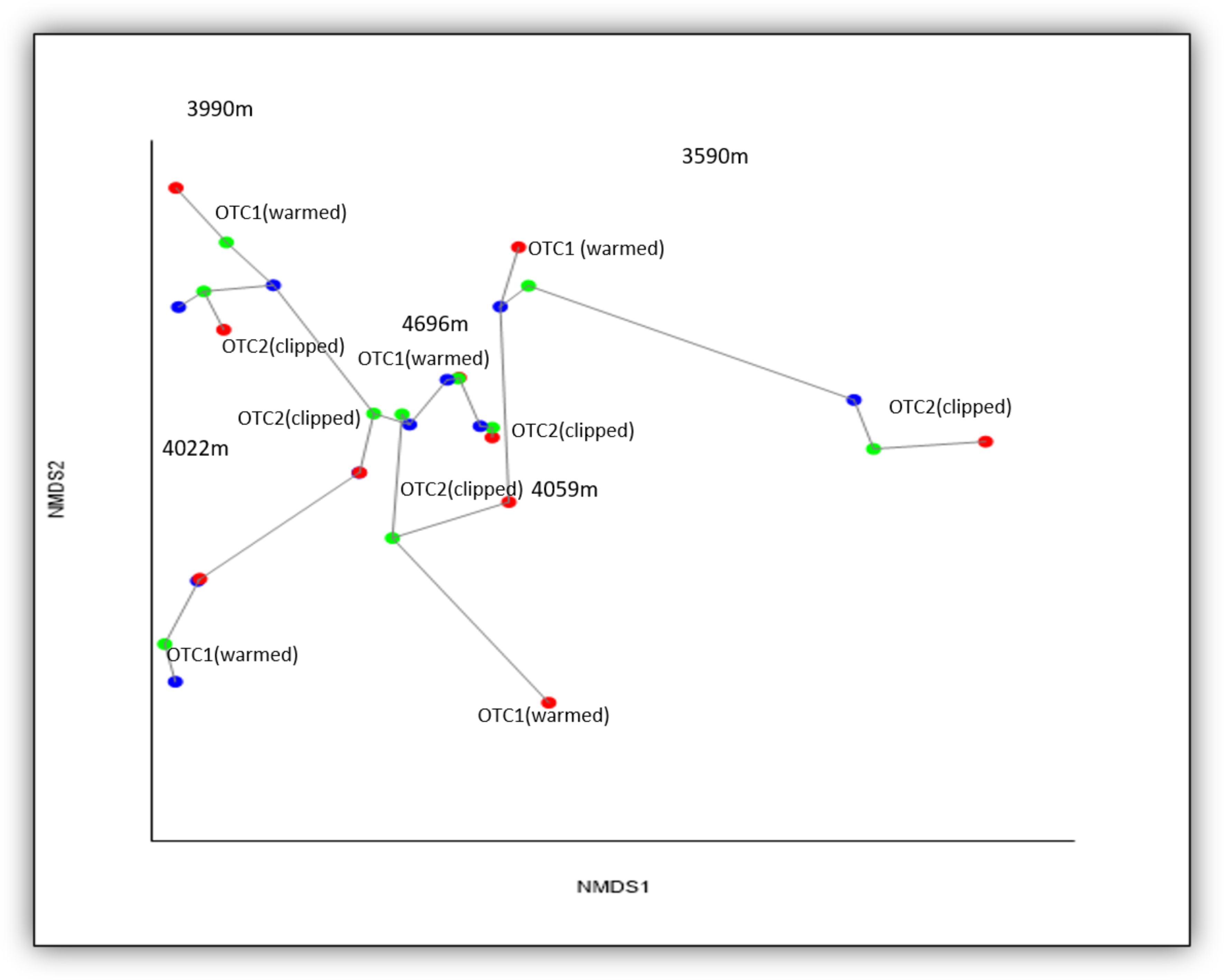

Supplement: S3 Fig — The relative abundance of plant species biomass inside the OTCs on each elevation site was estimated for all study years and shown by paths of mean values in Non-metric multi-dimensional scaling (NMDS) using Bray and Curtis dissimilarity index in R The plot shows no significant difference between the relative abundance of there is no significant change in the relative abundance of species inside both OTCs. OTC = Open top chamber, OTC1(warmed), OTC2, (warm*clip). Site 1 4,696m, Site 2 = 4,059m Site 3 = 4022m Site4 = 3,990m Site 5 = 3,590mDifferent color represents each study year, red, 2016, green, 2017, blue 2018. (TIFF) [file pone.0237893.s003.tiff]
