# Supplementary material for: The response of culturally important plants to experimental warming and clipping in Pakistan Himalayas
Source: PLoS One. 2021 May 6;16(5):e0237893. doi: 10.1371/journal.pone.0237893 (PMC8101745; doi:10.1371/journal.pone.0237893)
Supplement: S1 Table — Demographic features of informants interviewed for ethnobotanical information of culturally important plants. In the survey, their gender, age and socioeconomic details were recorded. A variety of plant species in experimental sites at different elevations. Each site was categorized according to the number of species present. majority of selected species were present at each site, but there was a representative specie of each elevation. Bistorta officnalis is present only at the highest altitude(4696m), similarly Plantago major (3690m) is present on site 5, lower altitude. (DOCX) [file pone.0237893.s004.docx]

**S1 Table. Demographic and Socio-Economic Characteristics of Informants**

| Interviewer | Type | Numbers |
| --- | --- | --- |
| Gender | Male | 53 |
|  | Female | 27 |
|  | Total | 80 |
| Age |  |  |
|  | Below 40 Years | 43 |
|  | Above 40 Years | 37 |
|  |  |  |
| Educational Profile |  |  |
|  | Ill- literate | 23 |
|  | Primary | 38 |
|  | Middle | 6 |
|  | High School | 5 |
|  | Graduate | 2 |
| Socio-Economic |  |  |
|  | Farmers | 6 |
|  | Policeman | 3 |
|  | Housewives | 11 |
|  | Forest Employees | 5 |
|  | Laborer | 15 |
|  | Councilor | 1 |
|  | Hakeem | 2 |

Demographic features of informants interviewed for ethnobotanical information of culturally important plants. In the survey, their gender, age and socioeconomical details were recorded.
