# Supplementary material for: The response of culturally important plants to experimental warming and clipping in Pakistan Himalayas
Source: PLoS One. 2021 May 6;16(5):e0237893. doi: 10.1371/journal.pone.0237893 (PMC8101745; doi:10.1371/journal.pone.0237893)
Supplement: S2 Table — A variety of plant species in experimental sites at different elevations. Each site was categorized according to the number of species present. Majority of selected species were present at each site, but there was a representative specie of each elevation. X” represents if the specie is present at one or all five sites. “X*” are selected plant species. (DOCX) [file pone.0237893.s005.docx]

| **Plant Species** | **Site 1**  **(4696m)** | **Site 2 (**  **4059m)** | **Site 3**  **(4022m)** | **Site 4**  **(3990m)** | **Site 5**  **(3590m)** |
| --- | --- | --- | --- | --- | --- |
| ***Artemisia rupestris*** |  | X | X | X | X |
| *Aster flaccidus* | X |  |  |  | X |
| *Astragalus penduncularis* | X | X |  | X |  |
| *Carex divisa* | X | X | X | X |  |
| *Smelowskia alba* | X | X | X | X |  |
| *Comastoma pulmonarium* | X | X |  |  |  |
| *Hedinia tibetica* |  | X | X |  |  |
| *Oxytropis glabra* |  | X |  | X | X |
| *Pedicularis cashmiriana* | X | X | X |  |  |
| *Poa alpina* | X | X | X | X | X |
| *Potentilla hololeuca* | X | X |  | X |  |
| *Primula macrophylla* | X | X | X |  | X |
| *Saussurea gnaphalodes* | X |  |  |  |  |
| *Silene gonosperma* | X | X | X | X |  |
| *Bistorta officinalis* | X |  |  |  |  |
| *Plantago major* |  |  |  |  | X |
| *Artemisia santolinifolia* |  |  |  | X | X |
| *Peganum harmala* |  |  |  | X | X |
| *Taraxacum afficinale* |  |  | X | X | X |
| *Temaricaria elegans* | X |  |  |  |  |
| *Myricaria elegans* | X |  |  |  |  |
| *Chenopodium foliosum* | X | X |  |  |  |
| *Draba oreades* | X | X |  |  |  |
| *Ciminalis aquatica* | X |  |  |  |  |

**S2 Table. Plant species composition and occurrence at experimental sites**

“X” represents if the specie is present at one or all five sites. A variety of plant species in experimental sites at different elevations. Each site was categorized according to the number of species present. Majority of selected species were present at each site, but there was a representative specie of each elevation. *Bistorta officinalis* is present only at highest altitude(4696m), similarly *Plantago major* (3690m) is present on site 5, lower altitude
