# Supplementary material for: The response of culturally important plants to experimental warming and clipping in Pakistan Himalayas
Source: PLoS One. 2021 May 6;16(5):e0237893. doi: 10.1371/journal.pone.0237893 (PMC8101745; doi:10.1371/journal.pone.0237893)
Supplement: S3 Table — a: Effect of warming on species percentage cover and biomass within the treatment. One way-ANOVA summary representing the individual percent increase/decrease in cover and biomass and the significant difference between the response of each species within the treatment. Significance codes: 0 ‘***’ 0.001 ‘**’ 0.01 ‘*’ 0.05 ‘.’ 0.1 ‘ ‘ 1. (b) Linear effect model summary of treatment, site and species effect on percentage cover and biomass. Linear mixed-effect model summary for each factor considered as fixed to evaluate the overall effect on aboveground biomass and percentage cover. Significance codes: 0 ‘***’ 0.001 ‘**’ 0.01 ‘*’ 0.05 ‘.’ 0.1 ‘‘ 1. (c) Summary statistics of mixed effect model for the significant effect of treatment, site, and species on percentage cover. (d) Summary statistics of mixed effect model for the significant effect of treatment, site, and species on aboveground biomass. (DOCX) [file pone.0237893.s006.docx]

**S3a Table. Effect of warming on species percentage cover and biomass within the treatment**

| Plant Species | Warming | | Clipping | |
| --- | --- | --- | --- | --- |
|  | **Percentage cover** | **Aboveground Biomass** | **Percentage cover** | **Aboveground Biomass** |
| *Poa alpina* | 18.7±4.29^***^ | 3.739±0.82 | -4.7±2.63 | -2.66±2.01 |
| *Artemisia rupestris* | 7.9±2.46* | 4.140 ±1.04 | -0.76±0.51 | 1.78±0.46 |
| *Potentilla hololeuca* | 8.5±3.17^*^ | 3.466±0.97 | -4.36±3.47 | 0.16±0.69 |
| *Pedicularis kashmiriana* | 7.3±2.44^*^ | 2.795±0.76 | -1.65±1.51 | -1.34±1.22 |
| *Astragalus penduncularis* | 9.8±4.05^*^ | 1.646±1.35 | -4.19±2.1 | -1.22±0.88 |
| *Silene gonosperma* | 6.8±2.1^**^ | 1.225±0.87 | -0.37±1.843 | 0.7±0.7 |
| *Smelowskia alba* | 6.1±2.20^**^ | 1.1±0.87 | -2.37±2.80 | -0.4±0.67 |
| *Artemisa rutifolia* | 7.1±2.88^**^ | 1.1±0.76 | 0.503±1.3 | -0.02±0.64 |
| *Oxytropis glabra* | 5.2±2.17^**^ | 1.04±0.94 | -2.42±2.31 | -0.42±0.44 |
| *Comastoma pulmonarium* | 2.8±1.19^**^ | 0.721±0.50 | -1.1±1.48 | -0.96±0.81 |
| *Carex divisa* | 2.6±1.59^**^ | 0.46±0.46 | -1.42±1.36 | -0.62±0.75 |
| *Taraxacum afficinale* | 2.8±1.44^*^ | 0.402±0.26 | -1.13±0.7 | 0.2±0.76 |
| *Primula macrophylla* | 2.6±1.73^*^ | 0.366±1.28 | 0.612±0.5 | -0.86±0.57 |
| *Plantago major* | 3±2.49^*^ | 0.109±0.56 | -0.87±1.4 | 0.78±0.78 |
| *Peganum harmala* | 1±0.66^*^ | -0.2±1.11 | -0.68±1.1 | 1.02±0.56 |
| *Bistorta officinalis* | 1±0.68^*^ | -0.182±0.18 | 0.210±0.29 | -0.4±0.45 |
| *Hedinia tibetica* | 2±1.33^*^ | 1.7±1.1 | 1.05±1.06 | -0.4±0.67 |
| *Saxifraga spp* | 1.5 | \| 0.72±0.50 \| \| --- \| \|  \| | -0.372±0.6 | -1.48±0.90 |
| *Total percentage increase/decrease* | 5.6% | 1.29% | -2.25% | -0.33% |

One way-ANOVA summary representing the individual percent increase/decrease in cover and biomass and the significant difference between the response of each species within the treatment. Significance codes: 0 ‘***’ 0.001 ‘**’ 0.01 ‘*’ 0.05 ‘.’ 0.1 ‘ ’ 1

**S3b Table. Linear effect model summary of treatment, site and species effect on percentage cover and biomass**

| Predictors | Percentage Cover | | Aboveground Biomass | |
| --- | --- | --- | --- | --- |
|  | **F-value** | **Pr(>F)** | **F- value** | **Pr(>F)** |
| Treatment | 15.0207 | 3.654e-09 *** | 35.4750 | <2e-16 ***  P<0.01 |
| Site | 6.3322 | 6.515e-05 *** | 1.8712 | 0.5331 |
| Species | 5.5997 | 1.308e-10 *** | 5.0575 | 2.332e-09 ***  P<0.01 |

*Linear mixed-effect model summary for each factor considered as fixed to evaluate the overall effect on aboveground biomass and percentage cover. Significance codes: 0 ‘***’ 0.001 ‘**’ 0.01 ‘*’ 0.05 ‘.’ 0.1 ‘ ’ 1*

**Factors: treatments*

**S3c Table. Summary statistics of mixed effect model for the significant effect of treatment, site, and species on percentage cover**

| Factors | Estimate | Std. error | t-value | Pr(>\|t\|) |
| --- | --- | --- | --- | --- |
| FactorWarming | 7.6824 | 1.3737 | 5.593 | 4.84e-08 *** |
| FactorClipping | 1.6000 | 1.3737 | 1.165 | 0.244988 |
| FactorWarm*Clip | -0.4941 | 1.3737 | -0.360 | 0.719304 |
| SiteM1 | -3.4081 | 1.5354 | -3.108 | 0.000044*** |
| SiteM2 | -2.3971 | 1.5358 | -1.561 | 0.119573 |
| SiteM3 | -1.1176 | 1.5358 | -0.728 | 0.467316 |
| SiteM4 | -6.3088 | 1.5358 | -4.108 | 0.178923 |
| SiteM5 | -5.4559 | 1.5358 | -3.552 | 0.440751 |
| SpeciesCD | -13.0000 | 2.8319 | -4.591 | 6.39e-06 *** |
| SpeciesAS | -2.3000 | 2.8319 | -0.812 | 0.417296 |
| SpeciesCP | -14.1000 | 2.8319 | -4.979 | 1.05e-06 *** |
| SpeciesFR | -8.4000 | 2.8319 | -2.966 | 0.003244 ** |
| SpeciesHT | -13.2500 | 2.8319 | -4.679 | 4.28e-06 *** |
| SpeciesMC | -15.0000 | 2.8319 | -5.297 | 2.21e-07 *** |
| SpeciesOX | -10.1500 | 2.8319 | -3.584 | 0.000391 *** |
| SpeciesPA | -2.0000 | 2.8319 | -0.706 | 0.0480553* |
| SpeciesPC | -10.5000 | 2.8319 | -3.708 | 0.000247 *** |
| SpeciesPH | -15.8000 | 2.8319 | -5.579 | 5.19e-08 *** |
| SpeciesPM | -12.7500 | 2.8319 | -4.502 | 9.46e-06 *** |
| SpeciesPT | -5.6000 | 2.8319 | -1.977 | 0.048854 * |
| SpeciesSG | -9.4500 | 2.8319 | -3.337 | 0.000948 *** |
| SpeciesSW | -10.5000 | 2.8319 | -3.708 | 0.000247 *** |
| SpeciesTM | -12.6500 | 2.8319 | -4.467 | 1.11e-05 *** |
| Observations |  |  |  |  |
| R2 |  | 0.336 |  |  |
| AdjustedR2 |  | 0.288 |  |  |
| Residual Std.Error |  | 8.955 (df = 316) |  |  |
| F Statistic |  | 6.956*** | < 2.2e-16 |  |

*Significance codes: 0 ‘***’ 0.001 ‘**’ 0.01 ‘*’ 0.05 ‘.’ 0.1 ‘ ’ 1*

**S3d Table. Summary statistics of mixed effect model for the significant effect of treatment, site, and species on aboveground biomass**

| Factors | Estimate | Std. error | t-value | Pr(>\|t\|) |
| --- | --- | --- | --- | --- |
| FactorWarming | 6.6479 | 0.5729 | 6.368 | 6.78e-10 *** |
| FactorClipping | -1.6803 | 0.5729 | -2.933 | 0.003601 ** |
| FactorWarm*Clip | -1.1792 | 0.5729 | -2.058 | 0.040380 * |
| SiteM1 | -0.9900 | 0.1773 | 0.690 | 0.091954 |
| SiteM2 | -0.9868 | 1.5358 | -1.561 | 0.119573 |
| SiteM3 | -0.7618 | 0.6385 | -1.193 | 0.233726 |
| SiteM4 | -1.5187 | 0.6410 | -2.369 | 0.018426 * |
| SiteM5 | -1.5000 | 0.6385 | -2.349 | 0.019423 * |
| SpeciesCD | 3.3700 | 1.1773 | -2.863 | 0.004484 ** |
| SpeciesAS | -1.3050 | 1.1773 | -1.108 | 0.268499 |
| SpeciesCP | -4.0000 | 1.1773 | -3.398 | 0.000767 *** |
| SpeciesFR | -4.2850 | 1.1773 | -3.640 | 0.000319 *** |
| SpeciesHT | -2.4050 | 1.1773 | -2.043 | 0.041901 * |
| SpeciesMC | -4.7450 | 1.1773 | -4.030 | 6.99e-05 *** |
| SpeciesOX | -4.1100 | 1.1773 | -3.491 | 0.000550 *** |
| SpeciesPA | 1.9900 | 1.1773 | 1.690 | 0.091954. |
| SpeciesPC | -1.4650 | 1.1773 | -1.244 | 0.214282 |
| SpeciesPH | -3.9600 | 1.1773 | -3.364 | 0.000864 *** |
| SpeciesPM | -4.6150 | 1.1773 | -3.920 | 0.000109 *** |
| SpeciesPT | -2.7500 | 1.1773 | -2.336 | 0.020125 * |
| SpeciesSG | -3.5700 | 1.1773 | -3.032 | 0.002628 ** |
| SpeciesSW | -4.1800 | 1.1773 | -3.551 | 0.000443 *** |
| SpeciesTM | -5.0885 | 1.1930 | -4.265 | 2.64e-05 *** |
| Observations |  |  |  |  |
| R2 |  | 0.3821 |  |  |
| AdjustedR2 |  | 0.337 |  |  |
| Residual Std.Error |  | 8.955 (df = 316) |  |  |
| F Statistic |  | 8.471 | p-value: < 2.2e16 |  |

*Significance codes: 0 ‘***’ 0.001 ‘**’ 0.01 ‘*’ 0.05 ‘.’ 0.1 ‘ ’ 1*
