# Supplementary material for: The response of culturally important plants to experimental warming and clipping in Pakistan Himalayas
Source: PLoS One. 2021 May 6;16(5):e0237893. doi: 10.1371/journal.pone.0237893 (PMC8101745; doi:10.1371/journal.pone.0237893)
Supplement: S4 Table — Significance. codes: 0 ‘***’ 0.001 ‘**’ 0.01 ‘*’ 0.05 ‘.’ 0.1 ‘ ‘ 1. Marginal R2 value represent variance by fixed effects and conditional R2 explained variance by the entire model (fixed and random). (DOCX) [file pone.0237893.s007.docx]

S4 Table. Summary from fitting a linear mixed-effects model by restricted maximum likelihood, to predict the effect of species-specific response to each treatment.

| Predictors | Percentage Cover | Aboveground Biomass |
| --- | --- | --- |
| (Intercept) | **<0.001***** | **0.155411** |
| Factor [Warming] | **<0.001***** | **<0.001***** |
| Factor [Clipping] | 0.144 | 0.986 |
| Factor [Warm*Clip] | 0.0954 | 0.279 |
| Factor [Warming] *Species [BS] | **0.000115 ***** | **4.22e-05 ***** |
| Factor [Clipping] *Species [BS] | 0.514 | 0.401 |
| Factor [Warm*Clip] *Species [BS] | 0.095310**.** | 0.091231**.** |
| Factor [Warming] *Species [CD] | **0.002340 **** | **2.14e-05 ***** |
| Factor [Clipping] *Species [CD] | 0.794 | 0.539 |
| Factor [Warm*Clip] *Species [CD] | 0.794 | **0.024** |
| Factor [Warming] *Species [CP] | **0.000456 ***** | **2.17e-07 ***** |
| Factor [Clipping] *Species [CP] | 0.602 | 0.625 |
| Factor [Warm*Clip] *Species [CP] | 0.514 | 0.249 |
| Factor [Warming] *Species [FR] | **0.005608 **** | **2.06e-06 ***** |
| Factor [Clipping] *Species [FR] | 0.602 | 0.688 |
| Factor [Warm*Clip] *Species [FR] | 0.079936**.** | 0.259 |
| Factor [Warming] *Species [HT] | **0.000198 ***** | 0.001763 ** |
| Factor [Clipping] *Species [HT] | 0.095310. | 0.475 |
| Factor [Warm*Clip] *Species [HT] | 0.896 | 0.075688**.** |
| Factor [Warming] *Species [MC] | **0.000115 ***** | **3.56e-06 ***** |
| Factor [Clipping] *Species [MC] | 0.695 | 0.915 |
| Factor [Warm*Clip] *Species [MC] | 0.695 | 0.29 |
| Factor [Warming] *Species [OX] | 0.045521* | **9.92e-07 ***** |
| Factor [Clipping] *Species [OX] | 0.433 | 0.688 |
| Factor [Warm*Clip] *Species [OX] | 0.24 | 0.095495**.** |
| Factor [Warming] *Species [PA] | **0.00036***** | 0.025 |
| Factor [Clipping] *Species [PA] | 0.296 | 0.116 |
| Factor [Warm*Clip] *Species [PA] | 0.695 | 0.552 |
| Factor [Warming] *Species [PC] | **0.005608 **** | 0.017045 * |
| Factor [Clipping] *Species [PC] | 0.695 | 0.319 |
| Factor [Warm*Clip] *Species [PC] | 0.695 | 0.054644**.** |
| Factor [Warming] *Species [PH] | **0.006097 **** | **0.002139 **** |
| Factor [Clipping] *Species [PH] | 0.602 | 0.254 |
| Factor [Warm*Clip] *Species [PH] | 0.449 | 0.89 |
| Factor [Warming] *Species [PM] | **0.001475 **** | 1.68e-06 *** |
| Factor [Clipping] *Species [PM] | 0.896 | 0.812 |
| Factor [Warm*Clip] *Species [PM] | 0.095310. | 0.234 |
| Factor [Warming] *Species [PT] | 0.002802 ** | **0.000278 ***** |
| Factor [Clipping] *Species [PT] | 0.433 | 0.085786. |
| Factor [Warm*Clip] *Species [PT] | 0.676 | 0.129 |
| Factor [Warming] *Species [SG] | **0.001109 **** | **8.02e-07 ***** |
| Factor [Clipping] *Species [SG] | 0.896 | 0.965 |
| Factor [Warm*Clip] *Species [SG] | 0.917 | 0.363 |
| Factor [Warming] *Species [SW] | **0.037287 *** | 1.88e-05 *** |
| Factor [Clipping] *Species [SW] | 0.361 | 0.702 |
| Factor [Warm*Clip] *Species [SW] | 0.192 | 0.186 |
| Factor [Warming] *Species [TM] | **0.002802 **** | **4.62e-07 ***** |
| Factor [Clipping] Species [TM] | 0.514 | 0.716 |
| Factor [Warm*Clip] *Species [TM] | 0.638 | 0.092 |
| Factor [Warming] *Species [AS] | 0.051826**.** | 0.022347 * |
| Factor [Clipping] Species [AS] | 0.621 | 0.607 |
| Factor [Warm*Clip] *Species [AS] | 0.721 | **0.21** |
| Random Effects |  |  |
| τ00 Site | 5.72 | 12.73 |
| ICC | 0.07 | 0 |
| Observations | 5 | 5 |
| Marginal R2 / Conditional R2 | 0.349/0.271 | 0.546 / 0.555 |

*Factor: Treatment

SumSq MeanSq NumDF DenDF F value Pr(>F)

**Treatment**  3613.7 1204.58 3 335 18.725 12.981e-11 ***

**Species** 7185.0 449.06 16 335 6.980 7.058e-14 ***

**Treatment:Species** 3791.0 78.98 48 335 1.2277 0.1546

Signif. codes: 0 ‘***’ 0.001 ‘**’ 0.01 ‘*’ 0.05 ‘.’ 0.1 ‘ ’ 1
