# Supplementary material for: The response of culturally important plants to experimental warming and clipping in Pakistan Himalayas
Source: PLoS One. 2021 May 6;16(5):e0237893. doi: 10.1371/journal.pone.0237893 (PMC8101745; doi:10.1371/journal.pone.0237893)
Supplement: Attachment — Submitted filename: Response to reviewers.docx [file pone.0237893.s009.docx]

**Response to The Editor and Reviewer’s comments**

**Title: The Response of Culturally Important Plants to Experimental Warming and Clipping in Pakistan Himalayas**

**Authors:** Saira Karimi, Muhammad Ali Nawaz, Saadia Naseem, Ahmed Akrem, Olivier Dangles, Zahid Ali^1*^

**Article Type: Original Study**

**Dear Editor,**

I am highly thankful for the valuable inputs given by you and the reviewers for the improvement of our manuscript titled “The Response of Culturally Important Plants to Experimental Warming and Clipping in Pakistan Himalayas”

Below are the responses to all comments raised by the reviewers. We gratefully acknowledge the time taken to provide valuable feedbacks. The helpful comments of the reviewers have contributed commendably for the improvement of the manuscript. We have made all the suggested revisions and a comprehensive yet coherent response is formatted (see below the Comments bold, black and responses to the Editor and Reviewer’s comments in normal italic font, color blue). The revised manuscript is resubmitted here please.

It is pertinent to mention that all key review remarks in 1) the experimental design, 2) statistical analysis and 3) language and grammar; have been incorporated with utmost consideration.

Once again, on behalf of all authors, I thank you very much for your valuable inputs, support and look forward for kind consideration to publish this manuscript.

Best regards,

**Zahid Ali (Ph.D.)**

**Dr. rer. nat.**

**Department of Biosciences**

**COMSATS University Islamabad (CUI), Islamabad, Pakistan**

E. mail: [zahidali@comsats.edu.pk](mailto:zahidali@comsats.edu.pk)

**Editors comment**

**First, the description of methods has to be improved. It remains unclear how the experiment was designed, thus more details of the experimental design have to be added. For instance, were all the (sub-)plots located within enclosures but clipped to simulate grazing? Was it a two-factorial experiment, with the factors warming and clipping crossed? Clarifying these details is essential to evaluate the appropriateness of statistical methods and the interpretation of results as well as to understand the discussion and the conclusion drawn.**

**Second, assuming that it was a two-factorial experiment with replicates along an environmental gradient, the statistical analyses (just comparing species within each treatment) would be inadequate. An appropriate analysis will be a two-factorial ANOVA including the main effects of warming and of clipping as well as the interaction between these two factors. This model would then, for instance, allow assessing if the effect of warming depends on whether vegetation was clipped or not (reflected by the interaction term), which is an important scientific question itself. Accordingly, results of the warming x grazing treatment have to be reported and discussed as well. Furthermore, if you consider the site effect to reflect some random variation, you should use a mixed model and include site as random effect. On the other hand, if you are interested in differences between sites, i.e. if you have a specific hypothesis how sites should differ or how the effects of clipping and warming should change along your environmental gradient, you may even include site and its interactions as fixed effects! If you conducted repeated measurements on the same plots, you could either average your data across census dates or should apply a repeated measure ANOVA. The two reviews provide many helpful suggestions how to improve your statistical analyses.**

**In general, the writing is a bit confusing and vague. The text has to be restructured in parts based on the comments by the two reviewers, and has to be carefully edited for grammar. The two reviewers provided many additional comments and detailed suggestions that have to be considered in a revised version of your manuscript.**

***Response to Editor*’*s 1^st^ comment***

*The treatments include warming (OTC1), clipping (simulated grazing), warming* x *clipping (OTC2) and control. All the measured plots were inside the enclosure, (also the subplot that was clipped).*

*Please see: Section Material and Method, page 9, line 192*

*Further details are described below as well*

*Warming treatment was employed using two six-sided open-top chambers (OTC) at each site.1.5* x *1.5m was divided into a central square region of 1* x*1 m dimension and six surrounding sloping regions. The central region was used for plant species composition sampling and the area under sloping sides of OTCs for destructive sampling (biomass and plant functional trait estimation). For each data point (OTC, clipped, warmed*clipped, control) the quadrat of 1m^2^ was placed and plant species cover, and biomass were assessed. The simulated grazing experiment consists of three comparisons: annual clipping plot inside the enclosure where the manual clipping of vegetation was done to the average height of vegetation outside the enclosure in an area that is grazed by herbivores and its control (un-warmed un-grazed) and OTC.*

*The diagrammatic experimental model of randomized block design (RBD) is given below.*

*At each site the field experiment unit (5x5m) comprised of a complete two by two factorial design( warming and clipping) with four treatments: warmed plots(OTC) clipped plots(CL), control plots and warmed plus clipped plot(W* x *CL). Each experimental unit of 5x5m is divided into subplot of 2.5* x *2.5 m. Open top chambers (OTCs bottom diameter 1.5m a top diameter of 1m were used to increase surface and air temperature since 2015. A 1* x *1m quadrat is placed randomly within each plot. This location was fixed for all sampling years and permanently marked. Percent cover of each species rooted within each quadrat was to be estimated once yearly, preferably at peak biomass. Second year of experiment onwards (after one full season of climate manipulation) above ground biomass was harvested and sorted out on species level, dried and weighed. Biomass was harvested within a plot but outside the 1m* x *1m quadrat. In OTC, it was under one of a six sides on rotational basis.*

**Editor’s comment 2**

**Second, assuming that it was a two-factorial experiment with replicates along an environmental gradient, the statistical analyses (just comparing species within each treatment) would be inadequate.** **An appropriate analysis will be a two-factorial ANOVA including the main effects of warming and of clipping as well as the interaction between these two factors. This model would then, for instance, allow assessing if the effect of warming depends on whether vegetation was clipped or not (reflected by the interaction term), which is an important scientific question itself. Accordingly, results of the warming x grazing treatment must be reported and discussed as well. Furthermore, if you consider the site effect to reflect some random variation, you should use a mixed model and include site as random effect. On the other hand, if you are interested in differences between sites, i.e. if you have a specific hypothesis how sites should differ or how the effects of clipping and warming should change along your environmental gradient, you may even include site and its interactions as fixed effects! If you conducted repeated measurements on the same plots, you could either average your data across census dates or should apply a repeated measure ANOVA. The two reviews provide many helpful suggestions how to improve your statistical analyses.In general, the writing is a bit confusing and vague. The text has to be restructured in parts based on the comments by the two reviewers, and has to be carefully edited for grammar. The two reviewers provided many additional comments and detailed suggestions that have to be considered in a revised version of your manuscript.**

***Response to Editor 2^nd^ comment***

*Statistical analysis has been improved keeping all the suggestions. We tried to improve our results and presentation to best of our data available in the guidance of editor and reviewers’ suggestions.*

*Please see: Section Material and Method, page 12, line 249*

*Further details are described below as well*

*The species-specific responses within each treatment and their significance was estimated through One-way ANOVA (S3 Table a&b). For each response factor (biomass, percentage cover), Two way ANOVA test was used to estimate the main and interactive effects of experimental warming and clipping. We constructed the linear mixed effect models (fit by maximum likelihood, package lmerMod) was used to determine the parsimonious relationships between the response factors and various biodiversity measures like treatments, plant diversity and elevational gradient (sites as random effect). We assumed the experimental treatments (warming (W), clipping (CL) and warming x clipping (W x CL)) and biodiversity measures (AGB, % cover) as fixed factors whereas sites and plant taxa firstly chosen as random factors and then only site as random factor to check the effect of each treatment per specie. Then susceptibility zones (Fig 4) were determined according to significant species responses depending on the interactive effects of both treatments (W x CL). To assess the change in the relative distribution of plant species within the plant communities, we used non-metric multidimensional scaling (NMDS) ordination analyses [58] on plant species that were present across the plot at each site (3590 m–4696 m) so that we could characterize plant community changes for each warming and grazing treatment groups. The Bray–Curtis dissimilarity measure was used to deal with relative abundance data as it allowed using both presence/absence and abundance data. All data analyses were carried out using the ‘vegan’ R package version 3.5.3 (2019).*

**Reviewer 1**

**Major comments**
1. General comment

**Comment:** **The experimental design is unclear. A diagram to include in or replace Figure 1 would help clarify this immensely. It was unclear whether all the measured plots were inside the grazing enclosures, or if any data was collected outside of the enclosures. It was also unclear whether there were four subplots in a factorial design (clipped, warmed, warmed + clipped, control) or some other nested design. Clarifying this is vital for readers to evaluate the statistical methods, interpret the results, and understand the discussion.**

***Response:***

*The diagrammatic experimental model of randomized block design (RBD) is given below.*

*Please see: Section Material and Method, page 9, line 192*

*Further details are described below as well*

*Each experimental unit of 5*x*5m is divided into subplot of 2.5* x *2.5 m. Where treatments were employed. The treatments were warmed (OTC1), clipped (simulated grazing), warmed*clipped (OTC2), and control. All the measured plots were inside the enclosure, (also the subplot that was clipped). Warming treatment was employed using two six-sided open-top chamber (OTC) at each site.1.5 x 1.5m was divided into a central square region of 1x1 m dimension and six surrounding regions. The central region was used for plant species composition sampling (1m^2^) and the area under sloping sides of OTCs for destructive sampling (biomass and plant functional trait estimation). For each data point (OTC, clipped, OTC*clipped, control) the quadrat of 1m^2^ were placed and plant species cover, and biomass were assessed.* *The simulated grazing experiment consists of two comparisons: annual clipping plot inside the enclosure where the manual clipping of vegetation was done to the average height of vegetation outside the enclosure in an area that is grazed by herbivores and its control (un-warmed un-grazed).*

**Comment:**

**The statistical methods do not seem to be appropriate. It is hard to suggest an exact method that would be appropriate since the experimental design is unclear. However, a two-way model with grazing and warming as separate factors (and potentially an interaction term as well, depending on design) would be a good start. Additionally, we suggest using a mixed-effects model with a random effect for site. The authors repeatedly note that site effects were stronger than the treatment effects and using a random effect for site will help separate these factors. Good packages in R for these types of simple mixed-effects models are 'lme4' using the lmer() function and 'nlme' using the nlme() function.

 For example, if there is no data collected outside the fences, and the experimental design is fully crossed warming and clipping (clipped, warmed, warmed + clipped, control), a good simple model specification (using lmer syntax) would be:
biomass ~ warming * clipping + (1|**[**site.id**](http://site.id/)**)
(which is the same as)
biomass ~ warming + clipping + warming: clipping + (1|**[**site.id**](http://site.id/)**)**
**This would then give you an output for the effect of warming, the effect of clipping, the interaction b etween warming and clipping, and with a random effect (intercept) for site identity (i.e.: site A, site B, ...) and you could run this model for each species individually. And similar separate models for cover.

There are more complex ways to approach this as well, including having all the species together in the model with either another random effect (intercept) for each species. However, if you think species respond differently to the different treatments, you will want a random slope, not just intercept. If you choose to go this route, or if there are also data collected outside the enclosure fences and you have a nested design, we recommend consulting a statistician to help with the model design. Again, clarifying the experimental design would really help with this.
An alternate approach if you think the differences between site are based on the altitude gradient, with predictable effects at high sites versus low sites, would be to use ANOVA with interaction terms (using the aov() function), and site altitude as a fixed predictor rather than site id as a random effect. The model specification could be:
biomass~ warming + clipping + site.alt + warming:clipping + warming:site.alt
although you might also choose to incorporate interaction between clipping and site altitude, or even a three-way interaction if you think that's appropriate.**

***Response:***

*I would like to appreciate the input given to do the analysis. We have followed each and every suggestion and re-analyzed our results using the Two-way ANOVA, and linear mixed effect models using fixed and random effects.*

*Please see: Section Material and Method, page 12, line 249,*

*Further details are described below as well*

*First the main and interactive effects of warming clipping and Warm* x *Clip were analyzed to have the evidence of significance of each factor and most importantly interaction effect on biomass and percent cover.*

*Please see section supplementary information (data summaries Table S3 Table a &b)*

*The data summaries of the model are added and described below as well*

*S3_Table(a)	The individual and interactive effect of warming, clipping and interaction between each treatment*

| ***Model*** | ***Aboveground Biomass (AGB)*** | ***Percentage Cover (PC)*** |
| --- | --- | --- |
| *Warming (W)* | *0.0000(< 2e-16 ***)* | *0.000(5.18e-14 ***)* |
| *Clipping (CL)* | *0.000315 **** | *0.583* |
| *WxCl.* | *0.014844 ** | *0.787* |

*Signif. codes: 0 ‘***’ 0.001 ‘**’ 0.01 ‘*’ 0.05 ‘.’ 0.1 ‘ ’ 1*

*Two-way Analysis of variance(2-ANOVA) was used to estimate the main and interactive effects of experimental Warming(W) and Clipping(CL) on the Above ground Biomass(AGB) and Percentage Cover(PC) across all experimental sites during the course of three experimental years(2016-2018). It summarizes individual effect of experimental warming exerted significant response in percentage cover and biomass while clipping’s negative response was not significant.*

*S3_Table(b)	Factor site and species effect on Percentage cover and Biomass*

| ***Variables*** | ***Perc Cover Significance*** | ***AGB_Significance*** |
| --- | --- | --- |
| ***Factors(W&CL)*** | *1.200e-06 ****  *P<0.01* | *<2e-16 ****  *P<0.01* |
| ***Site*** | *4.933e-05 ****  *P<0.01* | *0.5331* |
| ***Species*** | *.264e-10 ****  *P<0.01* | *<2e-16 ****  *P<0.01* |

Linear mixed effect model summary for each factor considered as fixed to evaluate the overall effect on biomass and cover

*Then linear mixed effect model were generated first to evaluate each factor response, determining the fixed (treatments) and random (site + species) and then fixed as (factors and species) keeping the site as random effect.(S3_Table a&b S5_Table c) The factor: species interaction was also analyzed to check species specific response to each treatment and has been graphically represented in the main figure 2B and supplementary figure S3_Figure A and B. The summary statistics of each model is provided in supplementary files(S3-S5Table)*

**S5 Table(a):** Summary statistics of linear mixed-effects model fit by maximum likelihood to determine the fixed and random effects of treatments, and site & species

| **Fixed Effects** | **Per-cover Significance** | **AGB Significance** |
| --- | --- | --- |
| Warming(W) | 0.000540*** | 4.05e-08 *** |
| Clipping (CL) | -0.000462 *** | -0.00625 ** |
| Warming and clipping(W*CL) | -4.09e-05 *** | -0.02992 * |
| **Random Effects** | **Marginal/Conditional R2 value** | **Marginal/Conditional R2 value** |
| Site and species | 0.151 / 0.318 | 0.205 / NA |
|  |  |  |

Signif. codes: 0 ‘***’ 0.001 ‘**’ 0.01 ‘*’ 0.05 ‘.’ 0.1 ‘ ’ 1. Marginal R^2^ value represent variance by fixed effects and conditional R^2^ explained variance by entire model (fixed and random)

The following figures are added in main manuscript as Fig 2 A&B

*Fig 2A: Linear mixed effect model representation of significant effects of each treatment (Fixed effect) on(A) percentage cover and (B) biomass. Warming significantly increased the Perc_Cover(A), while the combine effect of Warm and clip significantly decreased it suggesting that clipping has a negative effect on overall percent cover and biomass as compared to control. Random effect is not significant supporting the evidence that each experimental treatment effect is potentially affecting the biomass and cover and have strong evidences as compared to the altitudes (sites*)

**Fig 2B: Species specific responses towards each treatment by linear mixed effect model**

A) *Data showing the percent increase in cover of each species in control, warmed, and clipped plots* ***B)*** *significant change in aboveground biomass of each species in response to control, warmed and clipped plots*

*Other figures are added in supplementary section as S1 Fig-S4 Fig please refer to the supplementary section*

**Comment:**

**There are findings referred to in the discussion that are not included in the methods or results. These need to either be clearly incorporated as main parts of the paper or removed from the discussion. For example, flower production, analyses of community composition, and differences in response based on site location--see more detailed comments in the "discussion" section below.**

***Response:***

*The findings which are not included as main results have been removed*

*Please see: Section Discussion, page21, line 410,*

**Comment:**

**The discussion in general is not clearly organized. It would help a lot if you identified your 3-4 main results, and then gave each of these a paragraph in the discussion (then connecting it to existing literature as you have done).**
***Response:***

*Discussion is re-written included main results and structures keeping in view the valuable suggestions of reviewers*

*Please see: Section Discussion, page21, line 410,*

**Comment:**

**Please put the figure captions with the figures, it's very hard to evaluate it this way.**
*Response: Needful done*

**Comment:**

**The data summaries are included but none of the underlying data (the actually collected data points for each plot etc) is available in the supplements or stored in repositories (ex Dryad, Figshare, etc) online**

***Response:***

*The data files are submitted for online availability*

**Minor Comments:
Comment: Abstract: No information about any interaction between grazing and warming. Unclear if the experiment is set up to test this and they didn't find it, or if there were just the two treatments compared to one control.**

***Response:***

*Please refer to the abstract section page 1 line 16*

*which is re-phrased as*

*The relative effects of climate warming with grazing on medicinally important plants are not fully understood in HKH region. Therefore we combined the indigenous knowledge about culturally important therapeutic plants and climate change with experimental warming(OTC) and manual clipping (simulated grazing effect) and compare the relative difference on aboveground biomass and percent cover of plant species at five alpine meadow site on elevation gradient (4696m-3346m) from 2016-2018. There were significant responses towards main effect of experimental warming on biomass and percent cover (P<0.001) which increased throughout the plant community keeping the site as random. However, the interactive treatment effects (WxCL) were significant on biomass but not on percent cover. These responses were taxa specific. Warming induced an increase of 1±0.6% in Bistorta officinalis while for Poa alpina it was 18.7 ± 4.9% due to its abundance at the site. Contrastingly clipping has marginally significant effect in reducing the biomass and cover of all plant species. Simulated grazing treatment reduced vegetation cover & biomass by 2.3% and 6.26%, respectively, but that was not significant due to the high variability among taxa response at different sites. Thus, warming may increase the availability of therapeutic plants for indigenous people while overgrazing would have deteriorating effects locally. This research illustrates that vegetation sensitivity to warming and overgrazing is likely to affect man–environment relationships, and traditional knowledge on a regional scale.*

**Introduction**

**Comment**: **line 47 – 48 can you state a reference for this?**

***Response:***

*Please see section introduction page 3 line 49-51*

*Added the reference and sentence is re-structured*

In the Himalayas, an increase of 3.7 °C is expected in global mean surface temperatures between 2018–2100 (relative to 1986–2005). That is expected to cause substantial effects on the water cycle, biodiversity, and livelihood of the local population [8].

Kraaijenbrink PDA, Bierkens MFP, Lutz AF, Immerzeel WW. Impact of a global temperature rise of 1.5 degrees Celsius on Asia’s glaciers. Nature. 2017;549: 257–260. doi:10.1038/nature23878

**Comment
line 56 this reference is not specifically about grazing; can you find more suitable ones?**

**line 56 unclear what is meant with “selective palatability issues**

***Response***

*Added two suitable references as below.We have added this paragraph that better describes the term palatability, preference, and overgrazing relatedness.*

*Please see section introduction page 3 line 57-63*

*Further details are described below as well*

*The plant species selection by animals as feed is its preference. Grazing animals have sensory impulses stimulation that make them choose their preferred feed, either a plant or its parts which refers to the term palatability. This palatability is affected by animal as well as plant factors such as preference of forage species and seasonal availability of plant. Thus, some plants are selected repeatedly as forage which lead to exploitation by overgrazing. Overgrazing may lead to significant reduction in the palatable species and attribute to selective grazing of highly palatable species that are intolerant to severe grazing and flattening.*

*Kochare T, Tamir B, Kechero Y. Palatability and Animal Preferences of Plants in Small and Fragmented Land Holdings: The Case of Wolayta Zone, So*uthern Ethiopia. Agri Res Tech Open *Access J. 2018;14.*

*1Al-Rowaily SL, El-Bana MI, Al-Bakre DA, Assaeed AM, Hegazy AK, Ali MB. Effects of open grazing and livestock exclusion on floristic composition and diversity in natural ecosystem of Western Saudi Arabia. Saudi J Biol Sci. 2015;22: 430–437. doi:10.1016/j.sjbs.2015.04.012*

**Comment**

**line 64 you could start a new paragraph after “plant cover reduction”, this would facilitate reading the introduction and adds a clearer structure**

***Response***

*Needful done*

**Comment:**

**line 69-71 meaning unclear, For instance, as a result of extensive surveys to evaluate the nutritional status of higher altitude plants, it was proposed that higher altitude plants always had higher N content per unit leaf area when compare with contrasting altitudes a this increased with altitude in herbaceous plants [18]**

***Response:***

*Please see section introduction page 4 line 72-75*

*Further details are described below as well*

*Warming and grazing both have substantial impacts on the natural elements of high-altitude ecosystems, with critical consequences for local livelihoods [18]. In cold habitats such as the high Himalayas, leaves are generally rich in nutrients (high N content) to circumvent the detrimental effects of adverse environment on overall physiology of plants [19] These attributes and specific characteristics are making them more desirable and palatable for grazers* *[20].*

**Comment**

**line 75 has previous research dealt with overgrazing or simply with the impact of grazing?**

***Response:***

*yes, with warming and moderate grazing also with overgrazing the exploitation of natural biodiversity is studied*

**Comment**

**line 75 -76 add references to the first part of the sentence and specify that diversity in plants in general may be well covered by studies but not so information about medicinal plants. The way the sentence is phrased now does not convey the important message that studies on medicinal plants are still underrepresented**

***Response:***

*Please see section introduction page 4 line 81-84*

*Further details are described below as well*

*The paragraph is now structured as:*

*While a great deal of research has been conducted to predict warming and grazing effects on alpine biodiversity [20] , studies on medicinal plants are still lacking and indigenous knowledge is underrepresented where some indigenous communities rely, mostly on traditional system of medicines.*

**Comment: line 80-81 sentence unclear, explain “benefit-relevant indicators”**

***Response:***

*Please see section introduction page 5 line 86-90*

*Further details are described below as well*

*benefit relevant indicators are those measures that link any ecological and social outcomes in response to a change in the ecosystem. The paragraph has changed to explain the term more understandably*

**Comment line 83 – 84 you could delete this sentence, since it does not add important information**

***Response:***

*deleted*

**Comment: line 85 specify “resource” of what?, f.ex. income, remedies, etc.**

*Please see section introduction page 5 line 93- 95*

***Response:*** *the invaluable resources are herbal remedies prepared by local herbalists to earn their bread from also indigenous knowledge that transfers from generation to generations*

**Comment line: 86 "Second,"**

***Response:*** *improved the sentence structure*

**Comment: line 91--would be useful to give a rough metric of historical livestock vs current, instead of just saying it's increasing**

***Response:***

*no such data available though the report of pastures of KNP has rough estimation of >5000 potential grazers. The details are given in Material and methods 138-142*

**Comment: line 94--these two objectives seem pretty distinct, can you make the connection between them more clear?**

***Response:***

*Please see section introduction page 6 line 110-116*

*Further details are described below as well*

*The specific objectives of this study were to 1) indicate culturally and medicinally important plant communities responses to climate warming and grazing factors by manipulative experiments and 2) link these ecological factors to socioeconomic wellbeing of local inhabitants by assessing the dependence and utilization of these resources through ethnobotanical surveys. First to document the indigenous knowledge of culturally important medicinal plants through ethnobotanical surveys then to quantify the effects of climate change (warming) and grazing.*

**Comment:**

**I think in general the introduction could be improved by adding more structure and clarity by defining open questions/research gaps for each paragraph.
I also feel that references are partly missing or are not used in an accurate way**

***Response***

*the overall structure of introduction is updated as per your comments and suggestions. All the references are double checked for any missing one and style has been improve*d

**Comment : Materials and Methods**

**line 104 "July. "**

***Response***: corrected

**Comment: line 105 warmest year? warm years?**

***Response*:** *In a warm year, the summer temp in May has been reported up to 25*

**Comment: line 106 only few °C below zero or much colder?**

***Response:***

*Please see section Materials and Methods page 6 line 120-125*

*It was established in alpine zone where climate is defined by warm summers starting from May till August at some lower elevations (3340m) while at higher altitudes it ends in late early August. The maximum temperature in May in recorded the warm year goes up to 25ºC [41] while in winter it drops down much degrees below 0ºC (up to -10 ºC) from October (3590m) [15,42,43].*

**Comment:line 104-107 would be good to know temp and precipitation in the specific study years**

***Response*:** *It is explained further with more references about the trends of temperature increase in the regions and following paragraph is added*

*Please see section Materials and Methods page 6 line 126-130. Following references have been added*

*The current state of knowledge is based on the analyzed climatic trends in HKKH (Hindukush-Karakarm-Himalaya) region from data available since 1980 to 2009 [46]. indicate the rapid declines in the Great Himalaya which attributes to the global warming [47]. The reports of the weather station at Gilgit conclude during last 35 years there has been significant increase in mean annual temperature (average 0.65 ºC) which is higher than global average (0.17 ºC) [48].*

*35.	Bocchiola D, Diolaiuti G. Recent (1980-2009) evidence of climate change in the upper Karakoram, Pakistan. Theor Appl Climatol. 2013;113: 611–641. doi:10.1007/s00704-012-0803-y*

*36.	IPCC 2019. 2019 — Ipcc. 2019 [cited 1 Jun 2020]. Available: https://www.ipcc.ch/2019/*

*37.	Spies M. Mixed manifestations of climate change in high mountains: insights from a farming community in northern Pakistan. Clim Dev. 2019;0: 1–12. doi:10.1080/17565529.2019.170197*4

**Comment: line 110 "macrophylla, "**

***Response****: Corrected*

**Comment: line 112 "cattle and yaks" (although I recognize that cattles is also used in many countries, but it's the less common form)**

***Response****: Corrected*

**Comment: line 114 should be “Plantago lanceolata”
line 115 should be “Poa alpina”**

***Response:*** *Corrected*

**Comment:**

**line 117 greater than 5026 compared to what? And in what time frame and what is the unit (number of herbivores?)? Also 5026 is a very specific number, maybe just greater than**

**5000?**

***Response****:*

*Please see section Materials and Methods page 7 line 141-145*

*The summer pastures of KNP are subjected to grazing management system by indigenous communities because they are rich in herbaceous biomass [5]. In 2013, an analysis conducted to document the changing aspects of above ground biomass in high altitude alpine regions of Pakistan, proclaimed the number and types of livestock’s in KNP pastures featuring more than 5000 species including(sheep, goat cattle yak and others(horse &donkeys)[40].*

**Comment: Line 118 “immense burden” in what respect?**

***Response****:*

*Please see section Materials and Methods page 7 line 146*

*The structure of sentence is changed*

**Comment: line 119 varied rather than "the most versatile"**

***Response:*** *corrected*

**Comment:** line 119 - 120 need language edits in general

***Response:*** *Needful done*

**Comment:** line 128 "useful medicinal plant species are"

***Response****: corrected*

**Comment:** line 128 are these questionnaires accessible in the SI? If so, please refer to them.

***Response****: yes, needful done. These questionnaires are accessible in the SI.*

**Comment: line 119-126 this could be part of the introduction and would nicely support the aim of your study**

***Response****: added in introduction*

**Comment: line 129 could you add information on how these valleys were different? Given you study the impact of warming and grazing on specific medicinal plants it would be interesting to know the general species abundance of those plants in these valleys. We assume you chose these valleys because they combined people with medicinal plant knowledge and abundance of your studied species?**

***Response***
*yes, it’s what our hypothesis is based on. The purpose of conducting ethnobotanical survey was to gather the information about key medicinal plant species. How native people depend on them culturally and medicinally and to find about their abundance in the area. Then selection was based on the most cited plant species by informants and their occurrence on the experimental sites*

*For species abundance in these valleys’ study area has details of different vegetation zones in KNP and the representative species illustrating their abundance. Following paragraph is added*

*Please see section Materials and Methods; Ethnobotanical survey; page 7 line 152. Further details are below*

*Valleys of KNP are characterized by stony beds, water channels (Nullah), vegetation with grasses and large plants like bushes along slopes. Ghunjerab is covered with grasses and had pastures like appearance. Genus Artemisia exist here predominantly. In Shimshal valley, there are some agricultural zones as well and an abundance of sea buck thorn (Hippophae rhamnoides) crop. Plant species particular to family Asteraceae, Brassicaceae, and Eleagnaceae were cited considerably. Based on this initial screening survey, sites for manipulative experiments were designated comprised of the predominant species as excerpted by informants.*

**Comment: line 141 expression “collection of data the observations” unclear**

***Response****: Corrected*

**Comment: line 142--would be good to refer back to the altitudinal zones mentioned earlier--seems like this falls primarily into the middle zone (Alpine meadows)? If so, it may not be necessary to give such detail on the other two zones in the study site description.**

***Response:*** *Improved and un-necessary details are removed as below. The project included the installation of a manipulative experimental set up at five different sites along an altitudinal gradient ranging from 3590 to 4696 m which lies in alpine meadow of the KNP vegetation zones.*

**Comment: line 143 please also provide more details about the fence material you used i.e. height, mesh size, since this will inform the reader about the dimensions of the potential grazers in your system**

***Response****: The more details about mesh size and height of it is given.*

*Please see section Materials and Methods page 8 line 173*

*Each site consisted of a fenced area (un-grazed) of 5 x 5 m, which was designed as a randomized block design (RBD) and laid out four subplots of 2.5 x 2.5 m (Fig1).*

*We used standard cattle 1-2 wire fencing (1.7m height with 15x20cm mesh size).*

**Comment: line 148 your experimental set up seems to focus on summer warming only. Does this align with predications for your study area? Given that your fences allow smaller rodents to pass, how did the OTCs affect those and their impact on the vegetation?**

***Response****:*

*Yes, it focused on summer warming only because winters are very harsh, and these sites are covered in snow all over the winter thus access to the area is restricted. Also, in winter, the effect of manipulative warming would have not counted because of harsh weather, snow and wind*

*We included the investigation regarding summer temperature because all the understudy’ plants were perennial, also these pastures are accessible in summers only because KNP pastures at these elevations are summer pastures. Hence the effect of warming and clipping could be best studied during spring and summer.*

*Please see section Materials and Methods page 9 line 191-196*

*In general, the fenced area with OTCs acted as enclosure and grazing barrier for animals, however it did not eliminate small rodents and birds. But their effect on the plant destruction was not that significant because not only OTCs but as well as control and clipping plots were also subjected to their infringement. OTCs infact served as additional grazing barrier and they were not removed all the summer season. Grazing effect was induced by clipping only*

**Comment: line 149 does your observed temperature increase correspond with observations from other studies? Add references.**

***Response:***

*Please see section Materials and Methods page 10 line 197-199*

*It does correspond with the other studies as well as in line with the temperature projections by IPCC 2013. The warming effect of OTC at 10 cm above the soil surface was between 1.7–1.9°C on average[53,54].This temperature enhancement is within the range of expected degrees of warming in scenario of future climate change in the region[55].*

**Comment:: FIGURE 1 -- the photos are good but (a) should ideally show the grazing exclosures (if you have it) and it would be very useful to have a basic plot layout diagram to clarify the set up. It's unclear what photos d-g are supposed to show.**

***Response:***

*Figure 1 is removed and replaced by experimental design showing enclosures*

**Comment:**

l**ine 151 -- are the clipped subplots nested within the grazing enclosures? or within the open top chambers? Did you actually compare un-fenced areas, or is the clipping the only grazing treatment? That seems surprising since you have potential to do fenced and unfenced comparisons... would want better rationale for this decision. Herbivores can have many affects other than just clipping (dirt impaction, dung, nutrient cycling...)**

***Response:***

*Clipped plots were inside the fenced area. One clipped plot was warmed (OTC*clipping) as one of the two OTCs were clipped. The clipping was done to average height of vegetation in grazing plot outside fenced area which was used as reference to mimic the grazing impact. As there were management strategies about the grazing animals to use the KNP pastures our project funding did not included this factor. The unfenced comparisons were used only to mark the height of plants which were grazed upon. Comparison was done with control (fenced, no clipping, no herbivory) and outside the fenced area plot. Other nutrient addition treatment plot is not part of this study. Because it was not initiated in the first experimental year.*

*Please see section Materials and Methods page 10 line 200-214*

**Comment:**

**line 152-- no clipping, or no warming? So there were 2 warmed, 1 clipped, and 1 control? Or 2 warmed (1 clipped, 1 unclipped) and 2 unwarmed (1 clipped, 1 unclipped)?**

***Response:***

*Please see section Materials and Methods page 9 line 188*

*The treatments were warmed, clipped, warmed*clipped, and unclipped (control). All the measured plots were inside the grazing enclosure, (that was clipped).we can say 2 warmed( 1 clipped, 1 unclipped) and 2 unwarmed( 1 clipped and 1 unclipped)*

**Comment: line 153 vegetative height?**

***Response:*** *sapling height*

**Comment: line 154-156 unclear what this means, also how often did you clip the plants, does the clipping treatment mimic the natural grazing impact, did you mark the grazing level on individual plants? Please clarify what you have measured and if your measurements were done before or after the clipping and whether they were repeated during the peak season or several years.**

***Response:***

*The clipping treatment was done taking the reference of the grazing level of individual plants. Each year, we measured the height of individual plant and then we clipped and marked it according to the grazing level outside. Measurement were done before (length) and after the clipping and fresh weight was measured in field. After the samples were dried and measure for dry weight for biomass estimation of each species.*

*Please see section Materials and Methods page 10 line 201-214.*

*The experiment consisted of three treatments , a 2.5m plot outside fence for herbivory termed as Grazing, one similar dimensioned plot inside fence subjected to simulated clipping treatment in order to mimic the grazing treatment outside and their control(fenced).The clipping was done one time each year to effectively simulate growth.* *The simulated grazing experiment consists of two comparisons: annual clipping plot inside the enclosure where the manual clipping of vegetation was done to the average height of vegetation outside, the enclosure in an area that is grazed by herbivores and its control (un-warmed un-grazed). Plant height was measured, and clipping was done during the peak season each year (May–Aug). The grazing levels were permanently marked at the starting year and each plant species growth was recorded individually from the clipped point. In the clipped plots the aboveground part of all selected plant species were clipped to about 2-4cm on average above the plot. There was no disruption in the unclipped plots (OTC, control).* *Most of the selected plant species are perennials they grow well in growing season and shed off their seeds when it ends and sprouting begin at the start of spring each year.*

**Comment: line 169 the reference you state does not seem to focus on vegetation but on ground dwelling insects**

***Response:*** *corrected*

**Comment: line 171 what are the selected plant species?**

***Response****:*

*Please see section Materials and Methods page 11 line 227. Further details are added below as well.*

*The 17 plant species were selected among them are Artemisia rupestris (AR), Poa alpine (PA), Oxytropis glabra (OX), Plantago major (PM), Tamaricaria officinalis (TM), Comastoma pulmonarium (CP), Potentilla hololeucaI (PT), Carex divisa (CD), Astragulus penduncularis (AS), Silene gonospermum (SG), Smelowskia calycina (SW), Primula macrophylla (MC), Hedinia tibetica (HD), Saxifraga (FR), Pedicularis kashmiriana (PC), Bistorta officinalis (BS), Peganum hermala (PH)*.

**Comment:** l**ine 174 briefly explain this method in a short sub sentence**
***Response****:*

*Please see section Materials and Methods page 11 line 233. Further details are added below as well.*

*Implementing this method, we commonly measure the cover; defined as vertical projections of vegetation that include the area of a quadrat. In our study we used 1m^2^ quadrat for species sampling and cover estimation. The percentage cover of the selected plant species was recorded individually at the start of the experiment (2016) by visualizing the numbers of grids of quadrat covered by a plant species.*

**Comment:** **line 176 did you sample all occurring species or only those that you previously selecting. If the latter, why were they sampled for classification?**

***Response****:*

*Please see section Materials and Methods; Data sampling; page 10 line 236. Further details are added below as well.*

*Clipping and sampling was done each year, from both OTCs and Control plots. Inside OTC the 1m x 1m plot was designated for species cover and composition. the area along the slopping side was selected for destructive sampling(plant height + biomass).Aboveground biomass was sorted to species level and recorded carefully*
**Comment:** **line 179 What was the timing of the clipping relative to the plot surveys? Did you survey before or after clipping?**
***Response****: we surveyed in the initial year of experimentation. That was the first objective of the study. The clipping was done after the surveys of plots*
**Comment:** **line 180 why not a two-way ANOVA? are these treatments not interacting with each other?**

***Response****:*

*Please see section Materials and Methods page 12 line 249. Further details are added below as well.*

*We calculated the individual effect of warming (OTCs) and simulated grazing (clipping) on overall plant community and each selected plant species occurring within the experimental site by calculating the biomass and percentage cover of individual sapling.*
**Comment:** **line 181 If the treatments are interacting, it should be a two-way ANOVA. If they're not interacting, a Tukey test still doesn't seem like the best fit, since it's generally for different levels of the same type of treatments (ex-low intensity vs high intensity grazing). We have suggested a mixed-effects model as the best option (see major comment #2 above).
Given that you talk about “plant species” did you run separate models for each species?**

***Response****: Our observation consisted of a whole warming effect on plant community that includes these 17 species so aboveground biomass and percentage cover was estimated and model was run for each species where overall and individual response of each plant was estimated (using multiple comparison.Moreover, according to the suggestion mix effect model was run to evaluate the effect of fixed factors(treatments+Percentage cover and Biomass.*

*Please see section Materials and Methods page 12 line 252.*

**Comment: line 188 "The 'vegan' R package"
*Response****:* corrected

**Comment: Table 1 The inputs on what people have observed as climate change effects are really interesting! I think you could maybe highlight this more in your abstract/methods as a key component of the study. It would also be really interesting to directly compare the measured cover/biomass responses of species in the three categories: 1. observed by locals to have increased 2. observed by locals to have not changed in abundance 3. observed by locals to have decreased. Even if this is too small a sample to be statistically tested, it would be a great way to bridge the two components of the study. Also looking at the last two where the locals have observed range shifts--does this fit with the study's findings?**

***Response****:*

*There were reports by informants that many low elevation plants are now moving towards the high elevation and they mentioned the Tibet (Hedinia tibetica) quite astonishingly also Pedicularis kashmiriana is high elevation plant but its species observed at low elevation. It was an interesting fact that can link to current scenario of climate change. This supports the general change in specie phenology which is not directly related to this study but could be a supporting information of indigenous knowledge about local plants and future scenario. In discussion the relationship between our findings and survey observation is discussed.*

*Please see section Discussion page 21 line 406.*

**Comment: Is “no effect” and “not observed” the same? What does “forage availability” mean, i.e. due to climate change this species is now available for forage?**

***Response****:*

*Please see Table 1*

*The informants were asked questions about the overall vegetation and then about the plant species they use as medicine or is a representative of the area. Based on this information, then they were inquired about general changes in the availability of those species. They were well aware of the climate warming fact and confident enough to relate it to particular species which enhances the acceptance of the fact that these species are of high importance. Thus, their responses were categorized as no change (no effect), or any change that they might not have seen or not noticed about some species. Due to climate warming more pastures of KNP are now accessible, thus the locals perceived it as forage availability increased. Also, summer extension as main climate change impact, was mentioned and this observation included in a parallel study. Thus ultimately, summer extension may have increased the life cycle of certain plants thus they are present for long period.*
**Comment: Just out of curiousity: all species occur at quite high elevations, presumably with snow cover during winter, still some of them are available all year round (f. ex. flowers and leaves of Primula macrophylla) is there simply no snow cover or do these species prevail under the snow?**

***Response****: They were perennial the corrected*

**Comment: line 227 Saxifraga -- particular species?**

***Response****: This genus was not identified on species level*

**Comment: Fig 2. not really necessary, can just go into the table**

*Good suggestion. Added a column in the Table 1 with the name citation frequency (FC).*
**Fig 3. Looks like the warming and grazing treatments were crossed?
*Response****: One OTC was clipped and interaction between treatment is included in methods, results and discussion*
**Comment:** **line 237 All the more important to include site as a random factor in the model, also did you expect to see differences among warming treatments?**

***Response****: Site as random factor in model is included in statistical analysis*

*As there were two OTCs per site, we wanted to evaluate the distribution and relative abundance of each specie during the experimental years.*
**Comment: Line 240 is the grazing treatment the actual grazing treatment or the clipping treatment?**

***Response****: It is the clipping treatment referring to the simulated herbivory effect in the surroundings of fenced area.*

**Comment: line 259 This is where a two-way ANOVA with the interaction term would really show these results, not just having to visualize them graphically.**

***Response****: The mixed effect model suggested that the profound effect of warming has significantly increased the percentage cover of species. The analysis has shown the same effect and significance is added to Fig4*

*Please see section Results; Fig 4; page 21 line 400.*

**Comment: line 259-260 this is interesting and novel and should be emphasized more throughout the ms**

***Response****: We further assessed the response to experimental warming and grazing for the five plant species that were most cited by surveyed people (A. rupestris, Astragulus penduncularis, P.alpina, P.hololeuca and P macrophylla). These responses were divided into four categories: combined positive effects of warming and grazing (+W, +G), combined negative effects of warming and grazing (–W, –G) and antagonistic effects (–W, +G or +W, –G) (Fig 4, Table 1).*

**Comment: line 264 are these results from a model across species?**

***Response****: no, this is just graphical representation of susceptibility, but a cross model would make it statistically correct. This effect has already been shown in mixed effect model*

*As the results of individual treatment of warming and clipping, we selected most vulnerable species which were highly responsive to the treatment and being culturally important as mentioned in the survey. Based on occurrence of these species on each experimental site and to calculate the combined impact of warming and grazing, the model was run to check their response towards both warming and to put it more effectively we made four susceptibility zones to check the positive and negative interacting impacts of both treatments.*

**Comment: line 265 -269 what are the statistics for these results? Are they mentioned in the data analysis part? If not, please add!**

***Response****: Statistics were the same as cover and biomass of each species on each experimental site.*

*Please see section Materials and Methods page 12 line 255-260.*

**Comment: Figure 4 Are these the averages across sites? How do you define "susceptibility zones"? Please add more detail to the methods about this approach**

***Response****:*

*Please see section Results page 21 line 400. Also for methods please refer to section Materials and Methods line 258.*

*Yes, these were average across sites.*
**Comment: S2 Fig are the different colored points the three years? I think this would be clearer without the lines. Are the non-warmed plots here to compare to?
*Response****: yes, each color point is experimental year (2016-2018). non-warmed plots are not here. As we wanted to show the relative abundance of each species across the experimental years. The stats are included in the supplementary information (relative abundance).*

**Discussion
Comment: In general, try to line the discussion sections up with your main results, which will help with the organization of it. In the very beginning you can have a summary of your most important findings, which should not only focus on the ethnobotanical survey, but also on the warming/grazing results.
*Response*:** *The overall discussion has been improved and structured as per valuable comments and suggestion. The results are discussed in sections*
**Comment: line 294 – 296 have you tested this relationship?
*Response****: No, it is removed*
**Comment: line 297-299 this is repeated
*Response****: It is deleted*

**Comment: line 307 Did you analyze community composition overall? Or just visually with the NMDS? There is a function for this in the 'vegan' package, adonis() for PERMANOVA analysis that would be useful to support this part of your manuscript especially if it is one of your key findings.**

***Response****: We evaluated the relative abundance and tried to visualize with NMDS. It is not one of my key findings. We wanted to show the relative abundance by the collected point inside in response to warming in each year.*
**Comment: line 309 You haven't mentioned flower production as part of your methods or results--either remove this from the discussion or include it as part of your paper (i.e. in Methods and Results)**

***Response****: removed.*

**Comment: line 311 Where in your results section do you discuss differences between species responses to warming at different elevations? This would be really interesting, but it isn't clear.**

***Response****: Here, the results of warming are discussed as these species show positive response towards experimental warming by significantly increasing their cover it could be suggested that these species can survive at high altitudes in the onset of climate warming as our experimental sites are along the elevation gradient , and occurrence of these species at altitude and their positive response to the clipping and warming strongly supports the fact that they can adapt to the changing environment provided the controlled grazing.*

*Please see section Discussion page 25 line 491; Fig S2.*

**Comment: line 313-314 “new opportunities” this is quite a bold statement, maybe you can put it in context with medicinal plant use**

***Response****: Good comment. The whole structure of discussion has been changed accordingly. Please see Discussion part, page 21 onward*

**Comment: line 314 to improve the structure of your discussion you could start a new paragraph after ending with “… for people living under cold conditions”. Remove “while” from the beginning of the sentence, otherwise the sentence does not make sense**
***Response***: *Removed*

**Comment: line 318-319 as mentioned earlier it is unclear whether your data are from inside fences only or also from outside the fences. If you did not collect data from outside the fences than it does not make sense to argue with different cattle density. Please clarify this already in the Methods and adapt this sentence accordingly.**

***Response*:** *we used the unfenced area as a reference which could be used as a model to mimic the grazing effect. Also, the estimation of local herbivory suggested that KNP is rich in herbaceous biomass and an attractive place for local community as compared to the neighboring pastures (Ishaq et al., 2013). Knowing this fact, we proposed that if it continued to be the same situation, soon the flora of KNP will be under the grazing pressure. And will lose its biodiversity, as some species will adapt to this pressure (as shown in this study) other will not (negative impacts of grazing)*

**Comment: line 321 Here you mention cover collected outside cattle exclusion plots, but it's not clear from your methods or results that this data was collected and how it was collected/analyzed. Please either incorporate it fully or remove it from the discussion.**

***Response****: corrected as suggested*

*The inside plot (clipping) the impact of grazing did not influence the percentage cover significantly*

**Conclusion
Comment: line 329 “grasslands” is mentioned here for the first time, so it feels a bit out of place, maybe you can add “alpine”?**

***Response****: added*
**Comment: line 330 this sentence is nice, though we think an evaluation of ethnobotanical survey methods is not the focus of your study. As plant ecologist we suggest highlighting here plant responses to your experimental treatments and put that into context with the medicinal plants.**

***Response****: Conclusion is improved as per suggestion. Please see page 25-26*

*Please see section Conclusion page 25 line 507 onward.*

*Overall, our study is the first to provide experimental evidence at Khunjerab National Park, of the combined effect of warming and grazing on culturally important plants. Our results provide valuable information for the evaluation and prediction of alpine sensitivity to future threats. Linking indigenous knowledge with experimentation and evaluating the combined and interactive manipulation experiments are likely the advanced tools to examine the responses of alpine community which has proven by our studies it can provide a jumping pad to policymakers for designing mitigation and adaptation strategies for climate change in a region that is undergoing rapid change and for which scientific data are meager.*

**Reviewer #2:**

**Comment: This study addressed how culturally important alpine plants will respond to climate warming and increased grazing pressure. This is a relevant and understudied topic, and I appreciate the integration of experimental manipulations with data collected from interviews of local people. However, I was confused by the study design- were the grazing treatments simulated grazing (clipping) as stated in the methods (lines 151-152), or grazed by animals, as suggested in the discussion (lines 318-319)? There was mention of grazing enclosures, but it was not clear whether plots were established both within and outside of them. Also, how many plots were established at each site? A diagram of the different sites, plots, and subplots would be helpful in clarifying the study design. Also, were species responses averaged over all 5 sites? The authors state that there are large differences between the 5 sites along the elevational gradient, so it doesn’t seem appropriate to completely ignore this gradient. It seems that the 5 sites were treated as experimental replicates, but they are inherently different. Were there replicate plots at each site, as well?

In general, the methods and results could use much more detail (see line edits below). For instance, wasn’t there a warming x grazing treatment? I don’t see those results reported anywhere- just the separate effects of warming and grazing. Also, the discussion section could provide more synthesis about how the survey data and experimental results complement each other.

Overall, this is an interesting study (great work!), but the manuscript needs more detail and clarity. It also needs to be revised for grammar.**

**This study addressed how culturally important alpine plants will respond to climate warming and increased grazing pressure. This is a relevant and understudied topic, and I appreciate the integration of experimental manipulations with data collected from interviews of local people. However, I was confused by the study design- were the grazing treatments simulated grazing (clipping) as stated in the methods (lines 151-152), or grazed by animals, as suggested in the discussion (lines 318-319)? There was mention of grazing enclosures, but it was not clear whether plots were established both within and outside of them. Also, how many plots were established at each site? A diagram of the different sites, plots, and subplots would help clarify the study design. Also, were species responses averaged over all 5 sites? The authors state that there are large differences between the 5 sites along the elevational gradient, so it does not seem appropriate to completely ignore this gradient. It seems that the 5 sites were treated as experimental replicates, but they are inherently different. Were there replicate plots at each site, as well?**

***Response:***

*Please see section Material and Methods page 9 line 192. Further details are described below as well.*

*The treatments were warmed (OTC1), clipped (simulated grazing), warmed*clipped(OTC2), and control. All the measured plots were inside the enclosure, (also the subplot that was clipped).*

*Warming treatment was employed using two six-sided open-top chambers (OTC) at each site.1.5 x 1.5m was divided into a central square region of 1x1 m dimension and six surrounding sloping regions. The central region was used for plant species composition sampling and the area under sloping sides of OTCs for destructive sampling (biomass and plant functional trait estimation). For each data point (OTC, clipped, OTC*clipped, control) the quadrat of 1m^2^ was placed and plant species cover, and biomass were assessed. The simulated grazing experiment consists of three comparisons: annual clipping plot inside the enclosure where the manual clipping of vegetation was done to the average height of vegetation outside the enclosure in an area that is grazed by herbivores and its control (un-warmed un-grazed) and OTC.*

*The diagrammatic experimental model of randomized block design (RBD) is given below.*

*The grazing treatments are the simulated grazing (clipping) subplots at each site and herbivory effect was induced by manual clipping of vegetation to the average height of each plant species outside the fenced area serving as grazing treatment. Aboveground biomass was estimated by calculating the plant length before and after clipping and taking the fresh and dry weight of the clipped vegetation. At each site there was one clipping treatment with its control (no clipping-no warming) and a subplot outside the fenced area. The five sites are also experimental replicates.*

**Line edits:**

**Comment: Lines 25-26: The description of the treatments is confusing, and lists 2 separate “controls”
*Response*:**

*Please see section Abstract page 2 line 18-22.*

*Improved, there was only one control plot*
**Comment: Lines 30-31: Is the percentage cover or biomass reported here?
*Response***: Both *percentage cover or biomass reported here.*

*Please see section Abstract page 2 line 26-28.*

**Comment: Lines 67-68: how do nutrients “counteract” the harsh environment- need more explanation**
***Response****:*

*Please see section Introduction page 4 line 76-77. Further details are described below as well.*

*In cold habitats such as the high Himalayas, leaves are generally rich in nutrients (high N content) to circumvent the detrimental effects of adverse environment on overall physiology of plants [17] These attributes and specific characteristics are making them more desirable and palatable for grazers [18].*
**Comment: Lines 70-71: This is worded awkwardly
*Response:*** *improved*

*Please see section Introduction page 4 line 76-79.*

**Comment: Line 80: Please explain what “benefit-relevant indicators” are**

***Response****:*

*Please see section Introduction page 5 line 90. Further details are described below as well.*

*Benefit relevant indicators are those measures that link any ecological and social outcomes in response to a change in the ecosystem. The paragraph has changed to explain the term more understandably*

**Comment: Lines 128-137: Need much more detail about the surveys. What types of questions were asked? Were they oral or written? How were people selected for the survey?**

***Response****: The questionnaire was added in the supplementary data.*

*The designated team of snow leopard foundation had already communicated with the local community coordinators to inform and enlist people interested in the survey. We focused on elderly people because of their experience and indigenous knowledge. Demographic features are enlisted in supplementary information. Elderly informants’ Formal consent were taken from informants about data collection and publication. The questions were formulated with the purpose of procuring invaluable wealth to knowledge with special emphasis on medicinal and culturally important plants. Questions about important plants their local name, availability, part(s) used, disease treated, mode of preparation of drug, and other cultural used were asked. The questions were asked in local language taking the services of local interpreter*

*Please see section Materials and Methods page 7 line 151-168.*

**Comment: Lines 143-144: How do 2 plots fit inside a 5x5 m fenced area, when a single plot is 5x5 m (containing four 2.5x2.5 m subplots)?**

***Response****: Each site has a fenced area of 5* x *5m which is divided into 2.5* x *2.5m subplots designated to each treatment*

**Comment: Lines 151-152: The un-closed parentheses make this sentence confusing. What is considered a “control” in this experiment? It would be helpful to assign unique names to all your treatments so that you can remain consistent when referring to them.**

***Response****:*

*Please see section Materials and Methods page 9 line 196-204. Further details are described below as well.*

*Each experimental site has a warming treatment (OTC) and its control (un-warmed, unclipped subplot placed at 2m distance, clipping treatment (simulated grazing) and a warming x clipping treatment (OTC x Clipping) and a control.*

**Comment: Line 154: Does clipping (simulated grazing) one time each year effectively simulate the continuous season-long grazing this region usually experiences?**

***Response****:*

*Please see section Materials and Methods page 9 line 188-195. Further details are described below as well.*

*The experimental sites included summer pastures of alpine meadows. After fencing, the vegetation was observed in both fenced sites and surrounding open sites subjected to continuous grazing during the peak season. The herbaceous summer pastures of KNP are attributed to a grazing management system adapted by local community that allows them to bring their livestock for grazing during peak season which usually starts from late March and lasts till August. It is carefully observed and managed to control the repetitive grazing on same pastures. The grazing levels were permanently marked at the starting year and each plant species growth was recorded individually from the clipped point. All the selected plant species are perennials they grow well in growing season and shed off their seeds when it ends and sprouting begin at the start of spring each year. A summary of number and type of grazing animals in selected pastures of KNP was reported earlier making the sheep, yok, goats and cattle the potential grazers. [51].Thus controlled herds grazing and plant species being perennial effectively simulate the grazing effect.*

**Comment: Line 169: I know you cite a source for this, but it would be helpful to briefly describe the “quadrat sampling method.”**

***Response***:

*Please see section Materials and Methods; Data sampling page 11 line 231. Further details are described below as well.*

*Implementing this method, we commonly measure the cover; defined as vertical projections of vegetation that include the area of a quadrat. In our study we used 1m^2^ quadrat for species sampling and cover estimation. he percentage cover of the selected plant species was recorded individually at the start of the experiment (2016) by visualizing the numbers of grids of quadrat covered by a plant species.*

**Comemnt: Line 172: Size of the quadrat grids? Also, I thought the subplots were 2.5 x 2.5 m (not 1 x 1 m)?**

***Response****: Yes the plots were 2.5m the quadrat grid used were of 1m^2^*

**Comment: Lines 174-175: Plant height is measured, but not reported in the results.**

***Response****:*

*Please see section Materials and Methods page 12 line 244. Further details are described below as well.*

*The plant actual height, (length) its clipped length and clipped biomass were recorded each year and growth was measured as g/cm*
**Comment: Line 177: What does it mean that plants were “sampled”? Weren’t they just identified to species?**

***Response****: They were clipped for biomass estimation as well from clipped and OTC2 plot.*

**Comment: Lines 178-179: Were the entire plots clipped? And does repeated clipping of the same location for multiple years affect results? Are plant species mostly annuals or perennials? Were the control and grazing plots also clipped (only the OTC plots are mentioned).**

***Response:***

*Please see section Materials and Methods; Manipulative experimental design of warming and clipping; page 9 line 196-222. Further details are described below as well.*

*In OTCs, the central 1m x 1m portion was designated for plant species composition analysis, and cover estimation. the area around the sloping sides of OTC was consigned for destructive sampling. The entire plots were not clipped but used the quadrat sampling method, and after clipping the individual species were sorted for identification. Plant species were perennial, and their life cycle usually starts from April to August. The grazing plot inside the fenced area was clipped only. The control served as no warming and no grazing fenced subplot*

*The percentage of aboveground biomass and cover doesn’t seem to be affected by repeated the clipping treatment because they were sampled at peak of the growing seasons, each year, and the plant had the sufficient time to survive in presence of enough resources. Also the clipping was done inside the fence imposing a simulated grazing effect as in naturally grazed plot. There was no disruption in the unclipped plots (OTC, control).*

*In the clipped plots the aboveground parts of all selected plant species were clipped to about 2-4cm on average above the entire plot. Most of the selected plant species are perennials they grow well in the growing season and shed off their seeds when it ends and sprouting begins at the start of spring*

**Comment: Lines 180-181: It looks like the ANOVAs were just done to find differences between species within a single treatment (warming or grazing). However, you might want to compare the responses across the different treatments to determine if there are differences between plant growth in the control, grazing, warming, and grazing x warming plots. I find that a much more compelling question than differences between individual plant species. Also, it looks like you compared relative increases of the treatment plots compared to control plots (% increase/decrease), but this is not stated anywhere in your methods. Finally, are responses averaged over all 3 treatments years? Since you sampled the same plots multiple years in a row, you probably need to use a repeated measures ANOVA.**

***Response****: The responses were averaged over all 3 treatment years, the data used in Figure 3 was to evaluate the witn in treatment responses of each species towards main effect of warming and clipping and %increase and decrease calculated by the difference in control plot(2015) in first year and experimental plot in last experimental year(2018).The sampling was done annually each year during blooming season and control plot difference were also measured each year. The statistical analysis now has done using Two way ANOVA to estimate the main and interactive effects of treatment and linear mixed effect model to investigate the fixed and random effects of site, species and treatment.*

*Please see section Materials and Methods; Statistical analysis; page 12 line 253.*

**Comment: Lines 190-192: This sentence is not needed.**

***Response****: corrected*

**Comment: Lines 192-197: This should be included in the “Ethnobotanical Surveys” section of the Methods. It is out of place here.**

***Response****: moved to Material and Method*

**Comment: Lines 201-207: Need more details about the results of the surveys**

***Response:*** *Added the results of survey*

*Please see section Materials and Methods page 16 line 285-299. Further details are described below as well.*

*We interviewed a total of 80 informants who identified approximately 50 medicinal plant species widely spread in the area (see supplementary S1_ Table). Based on ethnobotanic survey results, some highly cited species were selected; those species are palatable, essential for therapeutic remedies and abundant on experimental site thus prone to the change in environment. Predominant botanical families comprised of Asteraceae, Fabaceae 8%, Solanaceae 6%, Tamaricaceae 4%, and others count for 2% or less (see Table 1). We have selected those characterized species that were cited by three or more informants, which is general criterion for reliability of medicinal plants uses[57]. Those interviews also allowed us to collect detailed information about the therapeutic properties of plant species along their altitudinal range of occurrence, time of blooming, part used for remedies, and potential climate change effect on their availability (Table 1).Based on the collected information, we screened out 17 highly important and common plant species, which we categorized based on their percentage of use Artemisia. rupestris (AR) was the most cited plant due to its availability and medicinal potential. Other widely cited plant genera include Poa alpina (PA) and Oxytropis glabra (OX), Taraxacum officinalis (TM), and Plantago major (PM)*

*In KNP therapeutic treatment is intended as an essential mode of primary health care to heal minor illness. The frequently reported and treated ailments were high blood pressure, diarrhea, skin allergies (40%), followed by eye irritation, fever, gastrointestinal diseases (25-30%) including stomachache, worms’ treatment, acidity (<30%) etc. Problems related to heart, diabetes, joint pains, obesity and metabolism were also reported.(see supplementary S1Figure).In our survey the promising reports of using medicinal plants to treat high blood pressure, gastrointestinal diseases, skin and eye allergies, cardio vascular disease could be due to prevalence of these disease but also the absence of effective and approachable pharmaceuticals. The relative citation frequency indicates the informant’s agreement was high for disease also the uniformity in usage of plant species.*

*The information related to other cultural uses of plant species one fundamental use as a feed and forage to livestock and wild animals As KNP is rich in herbaceous biomass, local communities rely on livestock to generate income. The summer pastures are important, and the mentioned species are frequently available there. Due to biodiversity, animals have preference for some species which is determined when livestock selectively nibbling off the new leaves of species. they do not usually feed upon the mature plant. A variety of methods were reported by informants to prepare remedies i.e. decoction, infusion, tincture, juice or ground to power etc. The dominant parts used in preparation of medicine were leaves, flowers or whole plant.*

**Comment:Line 213: I think a part was omitted since it starts in the middle of a sentence.
*Response****: Corrected*

**Comment: Line 234: Since you calculated % increase in the treatment plots compared to the control plots, how did you account for pre-existing variation in plant cover of the different species between the 2 plots?**

***Response****:*

*Each site selected for the experiment was relatively homogenous (in terms of*

*topography, substrate type, vegetation type,), dominated by herbaceous vegetation and representative of the ecosystem. Each site was thoroughly examined to account for*

*any variation in vegetation types, topographic or orographic factors. In the first experimental year, the species cover, count, abundance, the frequency was recorded before the estimation of the response.*

*Please see section Materials and Methods page 11 line 231. Further details are described below as well.*

**Comment: Lines 259-269: What data is used here? It looks like you are using separate plant responses to just the warming and just grazing plots. Where are the results for the warming x grazing plots?
*Response:*** *The interaction treatment results are included in linear mixed effect model. These results were to evaluate the intensity if response magnitude of each species and the difference between them*

*Please see section Materials and Methods page 12 line 260.*

**Comment: Line 295-296: How does the RFC value influence plant availability in the warming treatments? I thought RFC was just an indicator of how often people mentioned the species in the interviews.**
***Response****:*

*Please see section Discussion page 23 line 450. Further details are described below as well.*

*Here the sentence isn’t clearly structured (improved now). We wanted to build a relation between experimental evidences and survey information by quoting the similar results. This point is highlighted in main manuscript now so it is removed from here*
**Comment: Lines 297-299: These sentences are repeated.
*Response****: Removed the repeated sentences*
**Lines 309-310: This is the first mention of flower production- it is not reported in the methods or results. You can’t introduce new results in the discussion section.
*Response:*** *Removed the un-necessary details*
**Lines 323-324: “A constant increase in species cover through time can occur under moderate grazing conditions” --- Are there sources to back up this claim? Your results do not necessarily support this.
*Response****:*

*Conclusion is rephrased*

*Please see section Conclusion page 26 line 500.*

**Comment: Lines 334-338: Citations for this? Also, this would be good to mention earlier, maybe in the introduction.
*Response****: Removed from here and mentioned in introduction*

**Tables & Figures:
Comment: Table 1: You should specify which information is from the surveys and which is from another source (which should be cited). Also, how are the “most preferable” forage species determined?**

***Response:*** *The information related to other cultural uses of plant species one fundamental use as a feed and forage to livestock and wild animals As KNP is rich in herbaceous biomass, local communities rely on livestock to generate income. The summer pastures are important, and the mentioned species are frequently available there. Due to rich biodiversity, animals have preferences for some species which is determined when livestock selectively nibbling off the new leaves of that species. They do not usually feed upon the mature plant.*

*Please see section Results; Table 1 page 15 line 277 and 309-314. Further details are described below as well.*

**Comment: Fig 1**: **These pictures are lovely, but I think they are mostly unnecessary. Maybe just panel b showing the OTC. A better figure would be a diagram (or annotated picture) of your plots and study design.**
***Response****: corrected as per suggestion. Please see response 1*

**Comment: Fig 2: It might be nice to color the bars by plant functional group (shrub, forb, grass), so someone unfamiliar with the species can at least know the growth form.**

***Response:*** *Included the information in the Table to support the RFC value as suggested by the reviewer*
**Comment: Fig 3: The figures are very fuzzy, so it is hard to read, but I don’t see any letters showing statistical significance as stated in the legend. Also, what do the geometric shapes around each error bar show? You need units for the y-axis: Biomass (% change).
*Response****:*

*Please see section Results; Fig 3 page 20 line 383. Further details are described below as well.*

*The figure quality is improved and statistical significance is added. As only warmed plot showed the significant result of increase. These are pirate plots which has following components*. *Points show raw data, the geometric shape called bean show the smoothed density full data distribution. The biomass was calculated as growth g/cm the axis is labeled(plant height was measured before and after clipping and clipped biomass was weighed (fresh/dry)then growth was calculated as g/cm in initial year and g/cm in last experimental year)*
**Comment: Fig 4: What does each point represent? A different site? A different year? Also, similar to my comment above, what data is shown? I don’t see how you can report these 4 zones if just showing the response to the combined warming x grazing treatment.**

***Response****:*

*Please see section Results; Fig 4 page 21 line 408 and 309-314. Further details are described below as well.*

*Each point represents the %increased/decrease in species specific response to warming and clipping at different site. +W­-G means main effect of warming in absences of grazing.+W +G is the combined effect which represent the interaction between treatment and response of these species.*
**Comment: Fig S1: The colors in the pie chart (panel b) are impossible to distinguish- there are way too many categories. Consider an alternative format. Also, I’m unclear as to what is shown in that chart- is the “study area” across all 5 sites? And before or after the treatments? Doesn’t plant species coverage change significantly between the sites? Could be good to show separated by site.**

***Response****: The idea was to just illustrate the cited plant families, now this chart is removed and main families are reported in to main text*

*Please see section Results; page 16 line 289.*

**Comment: Fig S2: Isn’t one of the OTC “grazed” for each site? I would be helpful to specify which one is grazed for each site. Also, I assume each color is a different year of the study? If so, you need to add a legend.**
***Response****: yes, OTC2 was clipped, each color is the year of experiment. the legend is added*

*Please see section Supplementary information; Now as Fig S*

**S1 Table: In order to visualize which species are present across multiple sites, it would be better to list all possible species in a column on the left, and then put an “X” next to a species when it is present at a site. That way, a common species will have five “X’s” all the way across to indicate it’s present at all the sites.**

***Response****: Its done and described below as well.*

*Please see section Supplementary information; Now as Table S2*

**Comment: S3Table**: **You don’t need both columns- just include the mean ± SE column. Also, what values are reported? It looks like relative differences between the treatment and control plots (and not actual biomass (g/m2)). If so, this needs to be stated in the table legend. Is there also a table for the warming x grazing treatment?**

***Response:*** *Only one column of mean± SE is added. The %increase in cover and biomass , compared to control, in response to elevated warming, is recorded here. The objective was to demonstrate the within treatment species-wise variation to predict the occurrence. The interactive treatment effects are included in the main mixed effect model data summaries.*

*Please see section Supplementary information; Now as S4 Table*
