# Supplementary material for: The response of culturally important plants to experimental warming and clipping in Pakistan Himalayas
Source: PLoS One. 2021 May 6;16(5):e0237893. doi: 10.1371/journal.pone.0237893 (PMC8101745; doi:10.1371/journal.pone.0237893)
Supplement: Attachment — Submitted filename: Response to reviewer comments_2.docx [file pone.0237893.s010.docx]

**Authors response to the Editor and Reviewers**

**Title: The Response of Culturally Important Plants to Experimental Warming and Clipping in Pakistan Himalayas**

**Authors: Saira Karimi, Muhammad Ali Nawaz, Saadia Naseem, Ahmed Akrem, Olivier Dangles, Zahid Ali^1*^**

**Article Type: Original Study**

**Dear Editor,**

Please find enclosed the responses to all comments raised by the reviewers for our manuscript “The Response of Culturally Important Plants to Experimental Warming and Clipping in Pakistan Himalayas” to be considered for publication in PLOS-ONE. We gratefully acknowledge the time taken by the honorable reviewers to provide valuable feedback. The helpful comments of the reviewers have contributed commendably for the improvement of our manuscript. We have made all the suggested revisions and comprehensive, yet coherent responses are formatted (see below the responses to the Editor, and Reviewer’s comments in italics). It is pertinent to mention that all key review remarks in: 1) the statistical approaches have been narrowed down to recommended approach suitably, 2) The direction and storyline of the manuscript is defined further, and the connection of survey results to experimental pieces of evidence has been built-up and explained in a clearer fashion 3) the language and grammar have been improved further with the utmost consideration. Figures are also updated as suggested by both the reviewers. Here I would like to mention that about Figure 2, reviewer #1 accepted it as it is, in line graph and recommended minor edits. We have done all minor edits recommended by reviewer #1 for this Fig, however reviewer #2 suggested to change it into box and whiskers plot with suggestion that line graphs represent changes over time. It is humbly mentioned here that this statement supports to our study data too, we also studied climate warming impacts over a period of 3-years. Thus, we request to consider the Fig 2 in line graphical form. Figure 2 as box and whisker plot is also submitted as a separate file and with the rebuttal letter just for your perusal.

We have fulfilled all necessary requirements and I on behalf of all authors thank you very much, and request to kindly consider the manuscript for publication.

Sincerely,

**Zahid Ali (Ph.D.)**

**Dr. rer. nat.**

**Department of Biosciences**

**COMSATS University Islamabad, Islamabad campus**

**Pakistan**

E.mail: [zahidali@comsats.edu.pk](mailto:zahidali@comsats.edu.pk)

**Editors Comment 1: For instance, you applied a variety of different statistical approaches for the same research questions, which interprets results difficult and confusing. You may wish to focus on the approach that is most appropriate for a respective research question or hypothesis and show only tables and figures related to that particular approach. The reviewers provide various further recommendations on how to improve the clarity of figures and tables.**

**Response to Editor**

*We agree to this point about the statistical approaches that have been addressed in this revised version of the manuscript. Among all the analysis that were suggested by the respected editor and reviewers, the most appropriate analysis which describe our result most effectively is Linear effect model. The tables and figures related to only this model are shown and all other overlapping data is removed.*

*Please see statistical analysis in section Material and Methods page 11 line 228*

*result section see page 16 line 288*

**Comment 2:** I**n addition, the storyline, or direction, of your manuscript is still a bit unclear. For instance, you could have stronger focus on how your experimental results provide information about the future abundance of culturally important plants in your**

**region. Furthermore, I recommend considering the detailed recommendations and suggestions by the reviewers how to improve the introduction, the methods section, the results, and the discussion. Finally, the whole text would benefit from a thorough edit for grammar (including typos) as well as more clarity.**

**Response to Editor**

*We have clarified the focus of the manuscript and improved it in the direction which can relate our experimental results to the future abundance of medicinally important plants in the current scenario of climate change at Gilgit Baltistan. Moreover, the valuable comments and suggestions of both the reviewers have been considered and addressed carefully.*

*Please see section Discussion page 25 line 490-491*

**Reviewer 1**

**General Comment**

Our major suggestion is that you focus on one statistical approach and highlight the model output and related figures from that approach. At the moment you have added the approaches suggested by the reviewers, but as a result, have ended up with multiple overlapping modelling approaches. This makes the results difficult to interpret, and also an overwhelming number of supplemental tables and figures. The most appropriate one seems to us to be the linear mixed model with the random effects for site and species, which is discussed in the methods and highlighted in Figure 2 and Supplemental table S5a. Having both the ANOVA and the mixed model for the same response variables is redundant and confuses. In our following comments, we highlight ways that you can focus on and further clarify this statistical analysis and the results, as well as other more minor areas in the manuscript that need some revision*.*

**Response to General comment**

*This useful suggestion has been adapted and results of linear effect model are shown in Material and Method as well as results and discussion section All other overlapping information and statistical approaches (one-way ANOVA, two-way ANOVA results) are removed*

*Please see statistical analysis in section Material and Methods page 11 line 228*

*result section see page 16 line 288*

**Other general comments:**

**Plant height is mentioned in the methods as data you collected, and again in the discussion. However, it is not included in the statistical analysis methods or in the results. You either need to clearly explain how you analyzed height and what the results were or remove it from the manuscript. Check for typos in the main manuscript (species needs the 's' at the end for both one species and many species, authors names should be capitalized, other small typos) and the supplementary information**

***Response:***

*The plant height was used to measure the growth/cm along with biomass (see supplementary information data points) However, it is not the main result so has been removed. The typos and grammar mistakes have been carefully omitted.*

*Following key changes have been made in figures and Tables*

***Figures***

*Figure 1: More detail is added in the caption to best illustrate the experimental design and explain the sampling sites for species composition and biomass estimation. The schematic diagram has been improved with more clarification for plots and subplots.*

*Please see Section Results page 10 line 202*

**Comment:	Figure 2 seems to us to be the most useful results figure, and just needs some small edits. The subplots should be labeled A, B, C, D not A.A, A.B etc for clarity. Specify in the figure caption if the error bars show standard error, standard deviation, or something else. Also, the *** significance marks are not clear--do these show differences from the control? from each other?**

***Response*:**

*The subplots labels have been improved and added more details to it.*

*Please see Figure 2-page 18 line 317*

*The error bars show standard error. The significance marks represent the treatment’s significant effect over response variables as compared to control. The caption is edited as*

*Linear mixed effect model(lme) representation of significant effects of each treatment (fixed effect) on percentage cover and biomass. Warming significantly increased the percentage cover, while the combined effect of Warm and clip significantly decreased it suggesting that clipping has a negative effect on overall percent cover and biomass as compared to control.* ***a)*** *Percentage cover was higher in warming plots in comparison to control but Warm and clip treatment significantly had less cover of species suggesting that clipping has a negative effect on overall per cent cover.* ***b)*** *greater biomass of all species in warming treatment as compared to control and other treatments, suggested that under passive heating plants tend to increase their vegetative growth. The significant codes are 0 ‘***’ 0.001 ‘**’ 0.01 ‘*’ 0.05 ‘.’ 0.1 ‘ ’ 1.*

**Comment: Figure 3 this is mostly overlapping information with the second half of figure 2, and should be removed or moved to the supplement.**

***Response***

*It has been removed*

**Supplemental Figure S1 good as-is, although it would be clearer to write out "percentage" rather than "%age"**

***Response***

*The axis label is changed to percentage cover please see supplementary S1Figure.*

**Supplemental Figure S2 the order of the treatments in A and B is different, which is very confusing when looking quickly at the figure. Please change the order for S2B to match the order in Figure 2 and S2A. Please add more information to the figure caption with what tests etc were used to produce the figure.**

***Response***

*The order of the treatments is the same for both percentage cover and biomass, the caption has been improved as below. Please see supplementary information S2Fig*

***S2 Fig****: Species, Site and Treatment effects on percentage cover and biomass: a) the percentage cover of plant species increases in warming treatment and at multiple sites (M1, M2, M3). This is because of high abundance of these species on these sites while among species the response of individual species was not very different from each other, but some species responded more positively by increasing their percent cover as AR and AS. b) in comparison to control, above-ground biomass declined in clipping treatment but significant positive effect of warming treatment increased it for all species.*

**Reviewer comment:** **Supplemental Figure S3 is redundant with Figure 2 and can be removed**

***Response:*** *it is removed*

**Supplemental Figure S4 needs information in the figure caption as to what the different colors represent. Also, the caption says, "The relative abundance of plant species (presence/absence)" Which is it? Abundance measures are generally, biomass or maybe cover, while presence/absence is a binary.**

***Response***

*We acknowledge this point. S4figure updated as S3 figure also more details in caption are added. The relative abundance measure was biomass. The caption is improved as*

***S3Figure****:* ***Non-metric multidimensional scaling (NMDS) plot of plant species biomass with 95% confidence intervals using Bray and Curtis dissimilarity index over a 3 years experimental period along the elevation gradient of (3590–4696m).*** *The relative abundance of plant species biomass inside the OTCs on each elevation site was estimated for all study years and shown by paths of mean values in Non-metric multi-dimensional scaling (NMDS) using Bray and Curtis dissimilarity index in R The plot shows no significant difference between the relative abundance of there is no significant change in the relative abundance of species inside both OTCs.* *OTC = Open top chamber, OTC1(warmed), OTC2, (warm*clip). Site 1 4,696m, Site 2= 4,059m Site 3= 4022m Site4=3,990m Site 5= 3,590mDifferent color represents each study year, red, 2016, green, 2017, blue 2018*

**Reviewer’s comment: Tables**

**Supplemental table S2 This would be better to have one column for each site, and mark which species occur where rather than just tallying the number of sites they occur at. "Understudy" (used here and elsewhere in the ms) is unclear— do you mean understudied, that they have not been paid attention to in the literature?**

***Response***

*It has improved as per suggestion. Please see the section Supplementary information S2 Table. The caption is improved, and “understudy” is changed to selected plant species as follows.*

A *variety of plant species in experimental sites at different elevations. Each site was categorized according to the number of species present. Majority of selected species were present at each site, but there was a representative specie of each elevation.*

**Reviewer’s comment:**

**Supplemental Table S3 we recommend focusing on the linear mixed-effects model and dropping the ANOVA approach in the methods, results, and here. For that reason, we suggest deleting these tables.**

**Response:**

*These tables have been removed*

**Reviewer’s comment: Supplemental table S4 is good as it is**

***Response*: *Done***

**Reviewer’s comment: Supplemental table S5. We suggest moving Table a to the main manuscript and including F-values rather than just p values. These can be gotten by using the summary () function on the model fit. Table b should be dropped -- it's better to**

**have the interaction between the treatments than combining them in this way. Table c can be kept in the supplement.**

**Response:**

*S5Table a is moved to the main manuscript, which is now Table 2. Our model used t-test to assess the significance of the treatment, t-value are included in the main table, also the confidence level and estimates of the fixed effect to represent the best average effects.*

*Please see page 17- line 302*

I**ntroduction**

**Reviewer’s comment**

**Line 56 – 61 These information are all good, but go beyond scope of the introduction. From my point of view, the essential information would be contained in a sentence like “** **Repeated grazing can lead to exploitation and reduction of highly palatable species which are less resistant to severe grazing”. I still think that “selective palatability issue” is little unclear. Maybe instead of using this term you can rephrase it into the issues you are talking about. For example, do you mean that grazing leads to a change in forage species availability grazing animals can chose from? You could say “This leads to changes in plant availability that grazing animals can feed on and also to changes in nutrient availability”.**

**Nutrient availability of what? Leaves?**

**Response**

*Needful done. Please see Section Introduction page 3 line 57-62*

*Repeated grazing can initiate exploitation and reduction of highly palatable species which are less resistant to severe grazing. This leads to changes in plant availability and nutrient content to grazing animals feed and forage in natural ecosystems. Thus, some plants are selected repeatedly as forage an d causes the exploitation by overgrazing. It may cause a significant reduction in the palatable species and attribute to selective grazing of highly palatable species that are intolerant to severe grazing and flattening [11,12]*

**Line 85-86 Maybe you can add a reference to this sentence**

***Response:***

*Needful done. Following reference is added.*

*Olander LP, Johnston RJ, Tallis H, Kagan J, Maguire LA, Polasky S, et al. Benefit relevant indicators: Ecosystem services measures that link ecological and social outcomes. Ecol Indic. 2018;85: 1262–1272. doi:10.1016/j.ecolind.2017.12.001*

**Line 88-89 Even though you nicely improved this sentence, I still don’t have any idea about what these indicators are, maybe you can provide an example of such an indicator or explicitly write what are the indicators in your study. This information is essential for readers who have not been working with these kind of indicators before.**

***Response:***

Needful done. *The sentence has been simplified, please see the section Introduction page 5 line 85-88*

*To address these shortcomings, here, we provide an example of integrating plant community responses to grazing and warming with ecological and socioeconomics characteristics to assess how people's approach to culturally important plant species may be affected in the future.*

**Reviewer’s comment: Line 94 unique richness of what? Plants?**

***Response:*** *Unique plant species page 5 line 92*

**Reviewer’s comment: Line 108 you may mean “investigate” instead of “indicate”?**

***Response:*** *corrected page 5 line 105*

**Reviewer’s comment: Line 110 “factors” is not necessary.**

**Response:** *Corrected*

**Reviewer’s comment: Line 112 -114 This could be deleted since the information are already given in the objectives before. If you want to keep it, change the order so it matches the order of the objectives.**

**Response:** *Needful done,The repetitive information is deleted, and sentence structure is updated for more clarity. Please see the section Introduction page 5 line 104*

*Therefore, the specific objectives of this study were to 1) investigate culturally and medicinally important plant communities’ responses to climate warming and clipping (simulated grazing) by manipulative experiments and 2) to link these ecological factors to socioeconomic wellbeing of local inhabitants by assessing the dependence and utilization of these resources through ethnobotanical surveys.*

**Methods:**

**Reviewer’s comment: Line 119 km2, lowercase k**

**Response:** *Corrected page 6 line 113*

**Reviewer’s comment: Line 120 replace “it” with “the study”**

***Response:*** *Corrected page 6 line 114*

**Reviewer’s comment: Line 121 replace “it” with “summer”**

***Response:*** *Corrected line 115*

**Reviewer’s comment: Line 120-121 simplify this to just "from May till August" since it's also August at higher elevations.**

***Response:*** *Corrected line 115-117*

**Reviewer’s comment: Line 126 declines of what?**

***Response:*** *decline of glacier line 121*

**Reviewer’s comment: Line 129 "The alpine KNP region includes the following..."**

***Response:*** *Corrected line 125*

**Reviewer’s comment: Line 141 space missing between “biomass” and “(“**

***Response:*** *Corrected*

**Reviewer’s comment: Line 148 -151 The exact same sentences appear earlier in the introduction; you might want to delete them here to avoid unnecessary redundancy?**

***Response:*** *Thanks for pointing it. It is deleted*

**Reviewer’s comment: Line 153 Needs to refer to a specific table or part of the supplementary information (currently Supplement S6?). It may also be helpful to have one supplementary document with figures and tables and another one for the questionnaires only. This you could for example name supplementary information II, so the interested reader directly knows where to find the respective information.**

***Response:*** *The questionnaire is added as supplementary information S5. Please see supplementary information*

**Reviewer’s comment: Line 161 -163 This is phrased little awkward, I assume you mean that those species were abundant in your experimentalities? I suggest to rephrase this sentence.**

***Response:*** *the sentence is now rephrased Please see section Material and Methods page 7 line 146*

*Based on this initial screening survey, sites for manipulative experiments were selected where these species were in abundance as excerpted by the informants.*

**Reviewer’s comment**: **Line 163 This would be a good place to add more information on what main questions you asked about each plant, since most of your readers will not want to be searching through your supplement. Something like "For each species, we** **collected asked informants about... [climate change responses etc.]"**

**Response: *Needful done*.** *Please see section material and methods page 7 line 148-150*

*We asked questions about local medicinal plants, their traditional uses, methods of crude drug preparation, climate change responses etc.*

**Reviewers comment**: **Line 206 -209 I suggest that this entire part could be integrated the data sampling part**

***Response***

*Needful done: please see section material and methods page 10 line 209*

**Reviewer’s comment: Line 216 Why to stimulate growth, isn't the intention to simulate herbivory? Or do you mean you want to observe growth under herbivory?**

***Response:*** *Yes, it means growth under herbivory*

**Line 219 -220 For consistency it would be good to move the sentence about the height measurements to the data sampling paragraph**

***Response:*** *As plant height was measured before clipping and taking the saplings for biomass measurement, these results haven’t been included as main results, so we have removed this particular line about plant height measurement.*

**Line 257- 260 In the first part you write that you analyzed species-specific responses, but the tables show model results across species. I suggest you either adjust this in the text or refer to a table/graph with species-specific results. Furthermore, table b in S3 does report results of linear mixed effect models (according to the subscript of the table), this is confusing, because you don’t mention linear mixed effect models when you refer to the table in the supplementary information. Also related to table b in S3, it is not clear to me whether site and species were used as random or fixed factors in the model. In the text in your ms you report them as random factors, but in the table, they seem to be reported as fixed factors. Also, you only report the interaction of W x CL but no main effects. It would be good to include these in the table and also specify which test you used to assess the significance of the treatments like F- or ChiSq test. You can access these tests after you have run your mixed effect model via the Anova command from the “car”-package: Anova (mymodel, test.statistic = “F”) for F-test. Instead of using the summary statistics as you now did in table S5 you can instead report F-test results. Related to table S5 do you have an explanation for why the conditional R2results in NA when percentage cover is the response variable?**

**Response:**

*The statistical analysis section has now been improved Please see section material and methods page 11 line 228. The further response to the comment is as follows:*

*The species-specific response within each treatment and the variation of each species response towards the warming and clipping was estimated through one way ANOVA to investigate how significant was the response of a particular species (S3Table, Figure 3). Though the linear effect model also shows that concerning each treatment it is good to know the variance among species.*

*S3tables are removed as per reviewer’s comment. Results of the main and interactive treatment are included in Table 2 and S4Table. The model statistical summary where each treatment is considered as fixed with random effects of species and site is now added in the main text as Table2. The test we used for lme was t test and then to check the variance we used ANOVA chi sq test. The detailed statistics are also added in the table 2. The summary statistics of S5 table has been improved to remove the error of NA.*

**Line 261 Please add a reference to the R package you used. The most common packages to run linear mixed effect models are either lme4 (Bates et al. 2015) or nlme.**

***Response:*** *The reference is added. Please see page 11 line 234 [59]*

*59.Bates D, Mächler M, Bolker BM, Walker SC. Fitting linear mixed-effects models using lme4. J Stat Softw. 2015;67. doi:10.18637/jss.v067.i01*

**Reviewer’s comment: Line 264 -267 This is a key section that needs to be clarified. First, I think your response variables were the same as in your ANOVA models e.g. biomass and percentage cover, so these should not be termed as fixed factors but as response**

**variables. Second, neither biomass nor percentage cover is metrics of diversity! It can be helpful to include the model formulation here, which I think what you're trying to say is: cover ~ warming * clipping + (1|site) + (1|species). The best way to describe this model would be something like "Response variables of percentage cover and biomass were modelled using [xx package] with fixed effect predictors of the warming treatment, the clipping treatment, and their two-way interaction, and random effects for site and species (Table xx). We separately ran models with species as a fixed effect to check the effect of treatment for each species (Table xx)"**

***Response:***

*We agree to the reviewer’s viewpoint of keeping only linear mixed effect model results. The correction is made by removing the fixed factors but as response variables. Also, the model formulation is described, and a suitable reference is added by describing the details of the package used. The reference to the table showing the summary statistics is also mentioned as suggested by reviewers as follows also see material and methods page 11 line 231-240*

*The species-specific responses within each treatment and their significance were estimated through One-way ANOVA (S3 Table, S2Fig). Response variables of percentage cover and biomass were modelled using lme4 package [59] with fixed-effect predictors of the warming treatment, the clipping treatment and their two-way interaction(warming* x *clipping, W* x *CL ), and random effects for site and species(Table 2) We separately ran models with species as a fixed effect to check the effect of treatment for each species (S4Table) The following model formulation was used*

*Perc_cover ~ warming + Clipping+warming* clipping + (1|site) + (1|species) (main and interactive effect as fixed effect while site and species as random.*

*Perc_cover ~ Factor+species+Factor:species(1|site)*

**Reviewer’s comment: Line 267 I suggest starting a new paragraph for each different analysis you conducted--one for the linear mixed model, one for the susceptibility zones, one for the NMDS. I think you also need more information about this "susceptibility**

**zones" figure 4--why did you do it, how did you do it, what is it intended to show?**

**Response:A new paragraph is sectioned also more details are added to susceptibility zones**

*Please see section material and methods page 12 line 241*

*From the results of surveys (Table1) and linear effect model some highly medicinal and cultural important species (Artemisia rupetris, Poa alpina, Primula macrophylla, Potentilla hololeuca, Astragulus penduncularis) and illustrated their susceptibility to combine effect of warming and grazing (W* x *CL). The approach adopted was polygons-based susceptibility zones to show the positive and negative effects of both the treatments and future prediction about the occurrence of constitutive species under the stress of climate warming and herbivory.*

**Line 275** **You should also report which version of R or RStudio you used (latest version in R is 4.0.3). You can f.ex. write: We used R (RStudio) to run all analyses (R Core Team 2020 version XXX). For the vegan package you have to cite (****Oksanen et al. 2017). There is a neat function in R called citation() which shows you how to cite the packages you used**

**Response:**

*We highly appreciate reviewer’s helpful remarks and suggestion, the details of the version of RStudio with suitable reference is added. Sufficient information about susceptibility zones made under combine effects of both the treatments is added. Please see section M&M page 12 line 252*

A*ll the analyses were performed in RStudio 2.4-4. (R Core Team 2017) using the vegan packages [61]*

**Results**

**Reviewer’s comment: Line 321 - 328 This section with the ANOVA can be removed if you focus on the results from the linear mixed effect model. Also the numbers reported here don't match the p-values in S3 Table B, shouldn't they be consistent with that?**

***Response:*** *Needful done*

**Reviewers comment: Line 331 - 343 The language here makes it sound like you are testing the effect of the site "impact" rather than just variation. Also words like "increased" and "changes" make it sound like you're looking at the change through time, but your methods indicate you're just looking at the differences between treatments. Instead you should use statements like"has higher cover than the control" to match the analyses you're doing.**

**Response:**

*The result section has been updated accordingly. Please see section Results page 16 line 291-339*

*Further details are added as followed*

*The individual factor impact on the percentage cover and biomass is shown in the Fig 2 Table 2, illustrating the profound impact of warming(W), clipping (CL) and interaction (W x CL) between the treatments keeping the site as a random effect. Also, the overall differences in percentage cover of control and treatment plots at site MERGE1(M1, 4696 m) and site MERGE5(M5,3346 m), is found to be significantly different (M1 has higher cover and biomass than control while at M5 there is no significant variation between control and treatments(S2Fig)*. *At other sites (M2,4059 m, M3,4022 m, M4,3990 m) the response of species between control and treatment plots was similar (S2 Fig)*. *While species-specific response can be seen in species effect plot where some plant species as Poa alpina (PA), Astragulus penduncularis (AS), Artemisia rupestris (AR) Potentilla hololuca (PT) have significantly higher per cent cover in comparison to others while the above-ground biomass changes were significantly positive in the warmed plot of these species (S3Table,S2Fig). However, inter-annual variations among the species percentages cover were significant. Total increase in plant cover in response to warming was highly variable among taxa, ranging from 1 ± 0.6% for Bistorta officinalis to 18.7 ± 4.2% for Poa alpina (S3Table).*

**Reviewer’s comment: Line 337 It is advisable to avoid “seem” if you have statistical evidence for something affecting.**

***Response:*** *corrected*

**Reviewer’s comment: Line 340 Negative? I thought it was positive--higher in the warmed plots?**

***Response:*** *Corrected*

**Line 341 How can you state this, if you have pooled the data across years? Did you run separate models for separate years, or did you include the year as a fixed factor in your mixed effect model? You would need to either explain these models in your statistical analysis section and include them as ex supplemental tables or remove this part.**

**Response:** *The confusing sentence is removed*

*We pooled the data across the years and did not check the effect of year on growth instead considered the final experimental year as a result.*

**Line 344 You should have 1-2 more sentences here on the species-specific results that are presented in Figure 2**

**Response**: Needful done *Please see section results page 19 line 339-346*

*The plant community in warming plots responded significantly positive towards warming, while the response magnitude and intensity was highly variable among species. Important species - Artemisia rupestris Poa alpina, Carex divisa, Saxifraga, Taraxacum officinale etc have higher percentage cover and biomass in warmed plots in comparison to control and other treatment plots. Above ground biomass was also higher in warmed plots for some species. The negative effect of clipping and interactive treatment was evident for species such as Taraxacum officinale (TM) Peganum hermala (PH) and Primula macrophylla (PC) (Fig 3)*

**Line 347-351 This sentence is not clear to me. Do you intent to say that sites did not significantly increase the variation in your data? Assuming you had site as a random effect in your model.**

***Response:*** *While keeping the site as random, it was intended to state that site might be the determining factor for certain species significant response variables as considering the fixed factors the variability was high among site species responded differently at different sites.*

*Please see section results page 18 line 3**30-333*

**Commnent: Line 355 You can compare the responses in your treatment’s plots to the control, but usually control is not considered a treatment. It would thus sound better if you wrote something like "...****in above ground biomass of each species in response to warming, clipping and warming x clipping compared to control.**

***Response:* Needful done**

*Please see section results page 19 line 348 - 350. In above ground biomass of each species in response to warming, clipping, and warming* x *clipping compared to control, warming has overall positive effect on percentage cover and biomass of species while warming* x *clipping has negative effect. The * shows significant change in particular species with respect to the specific treatment as compared to control while the dots are outliners*

**Line 378 Should there be a new paragraph here from the NMDS results?**

***Response:*** *Corrected see page 20 line 365*

**Reviewer’s comment: Line 380 Isn't this supplemental figure S4?**

***Response:*** *Corrected*

**Reviewer’s comment: Line 382 This supports the choice to incorporate site as a random effect in your mixed effects model**

**Reviewer’s comment: Line 403 Delete “for”**

**Response:** *Corrected*

**Discussion**

**In your discussion, it would be nice if you could further emphasize the connection between your experimental findings and the ethnobotanical survey. Also, instead of AGB just say biomass, it doesn't take much more space and is much easier to read.**

**Response: *Needful done*.** *We have further elaborated the connection between our experimental results and survey information and justified the correlation. Please see section Discussion page 25 line 490-491*

**Reviewer’s comment: Line 418 You did not measure community data, but rather species-specific warming and clipping responses.**

**Response: *Needful done.*** *Please see section “Discussion” page 21 line 389-390*

**Line 419 - 420 The commas before and after "was" should be removed, and there should be a comma added after "sites"**

**Response:** *Corrected*

**Line 425-427 In your methods section (the statistical analysis) you don't report that you have used height as a response variable, nor do you report it in any of your results. If you did, you should add it, otherwise, you cannot discuss it here.**

**Response: *Needful done*** *paragraph from line 393-396*

**Line 429 I think you are combining two plausible mechanisms here: competition for resources and limitation in space. You might want to elaborate on both of them. Check ex. Borer et al. 2014 in Nature**

**Response:** *The mechanism details are given, and sentence structure is rephrased as follows also Please see page 21 line 402-412*

*This is attributable to increased competition, as biomass increased, plants were usually taller, and few individual species could fit into plots. Warming may lead to enhance plant growth and cover, it reduced the diversity of species as compared to the clipping treatment. In clipping treatment plots, the plant species did not grow well and had reduced their biomass and cover, but they maintain the diversity. This mechanism was elaborated by Borer et al, 2014[67] who demonstrated the effect of herbivory on the grassland production and showed that where herbivory increased the ground light it also maintains the biodiversity. Hence, where warming benefits some plant species in increasing their cover and biomass, it is likely to induce the dominancy of some species, whereas our experimental results suggested that clipping rescued the biodiversity.*

**Line 463 "Other studies on alpine meadows [where?] also suggested..."**

**Response:** *Norway, corrected and added reference Please see Discussion page 23 line 447*

**Line 469 Since intensity and frequency of clipping should have been the same for all of your studied species, I think it is rather something else that drives this species-specific response. You could think about whether this species has specific traits allowing it to regrow fast after clipping/grazing which the other species might not have.**

**Response:**

*Please see page 24 line 453-460*

*These different responses of vegetative biomass to clipping/simulated grazing can be attributed to specie- specific traits which allow the plant species to regrow faster than the others. Also, where clipping helped the certain plant species by increasing the ground level light, warming triggered the temperature and plant responded positively to the W* x *CL treatment. Some plants have evolved tolerance to herbivory by growing compensatory tissues so rapidly that help to cope with damage, thus allowing natural selection to favor herbivores and prompting plants to develop a new form of resistance, such as reduced apparency, chemical defenses (secondary metabolites) and indirect defense [73].*

**Line 473 "clipping" not grazing**

***Response:*** *Corrected*

**Line 478 "stronger" not "sturdy"?**

***Response:*** *Corrected*

**Line 490-492 This sentence is not clear to me. Do you want to state that the species you studied are more susceptible to warming than to grazing?**

***Response****: It is explained as follows: Please see page 25 line 480-482*

*They are vulnerable to clipping but warming has favored some species over others, so those selected species outcompete the other slow growers. The natural selection may play a role for these plants to be more abundant, thus palatable for grazers.*

**Line 493 Wang et al. add the year**

**Response:** *Wang et al., 2019 line 483*

**Line 494-499** **The first part of this part is about the impact of clipping, but you then refer to results of your survey in relation to warming. Please, clarify this. Also, can you state references for the loss of biodiversity. There are plenty of good ones available!**

**Response: new reference has been added and rephrased the paragraph as follow**

*Please refer to page 25 line 484-490*

*In our study, some of the species [P. alpina (PA), A. rupestris (AR)] responded significantly towards climate warming and clipping but not reducing their biomass in comparison to others (Fig 2). This supports the assumption of natural selection where strong competitor species surpass weak competitors, thus lead to loss of biodiversity[34,53]. This experimental evidence was also in line with the information gathered from the local informants, mentioning increased forage availability of these species throughout climate warming.*

**Line 501 You have not reported any measures of diversity related variables like species richness, so speaking about biodiversity isn't appropriate here.**

**Response:** *Our maximum plant species biodiversity was at site 1 and they responded positively towards warming. Here the purpose of explaining the dete/rments of biodiversity is to predict the species diversity over here in current scenario of climate warming*. *The species composition and diversity on experimental site is further elaborated.*

*Please see page 25 line 491-500*

**Reviewer 2**

**General comment**

**The authors have made substantial improvements, especially with describing the experimental design and survey methods. However, while there have been clear attempts to improve the statistical methods and results, I’m unclear about which approaches were used and why. It seems the authors used several different models (one-way ANOVA, two-way ANOVA, mixed models with different random factors), but I don’t think they are all necessary. The model interpretations in the results section are confusing, and do not clearly align with specific experimental questions. Instead, the authors should decide which questions are most interesting and choose 1-2 statistical methods based on that. Also, I’m unclear on how the % increase/decrease values were calculated (lines 250-252) and whether they represent the entire 3-year experimental duration. I still think it could be best to use repeated measures with year as a variable in the statistical model to see how the warming and clipping effects built up (or not) over multiple treatment years. Also, there is some discussion about differences between elevational plots (i.e., lines 333-335, 500-505) but it’s unclear how that was formally assessed.**

**Response:**

*The valuable comments and suggestions of reviewers have been adapted carefully and linear mixed effect models’ approach is used to answer the main and interactive effects of treatments while keeping the site and species as random effects. As we want to show the treatment effects on response variables of biomass and percentage cover of selected species, species-specific response to check the variation within treatment is also shown and interpreted.*

*During three-year course study (2015-2018) we have pooled the data across years to show main and interactive effects and did not run separate models for separate years as it was not the objective. We have not included a year as a fixed factor in our mixed effect model*. *The per cent increase and decrease value were calculated as considering first experimental year as control and last experimental year as the effect of treatment thus the values are then compared with control to determine the %increase or decrease in experimental plots. The random effect of the site is further explained through NMDS to check the variation in species relative abundance of biomass in the OTCs plots.*

*Please see statistical analysis in section Material and Methods page 11 line 228*

*result section see page 16 line 288*

**Reviewer’s Comment: Lines 44-46: This is unclear. Land and water degradation do not “cause” climate change.**

**Response:** *Please see section introduction page 3 line 44-46*

*The sentence is structured for more clarity. Importantly, high altitude regions are already under the stress of land and water degradation, climate change may exert extra pressure and makes them highly exposed*

**Line 49: Not “global” mean surface temperature if just referring to the Himalayan region.**

**Response:** *Corrected*

**Lines 85-86: I appreciate that “ecosystem change assessment” is defined, but the definition is still unclear. Also, the rest of the paragraph does not mention these “ecosystem change assessments,” so why is it brought up here?**

**Response:** *The lines have moved to start of the paragraph to give it a more clarity please see page 4 line 80*

**Reviewer’s comment: Line 106: “primarily runs the financial circle” is unclear**

***Response:*** *Removed from the text as it is irrelevant here*

**Reviewer’s comment:** **Lines 112-114: This last sentence is unnecessary**

***Response:*** *Removed*

**Line 123: Incomplete sentence**

***Response:*** *please see section M&M page 6 line 115-117*

*Corrected as : The maximum temperature in May recorded goes up to 25ºC [44] while in winter it drops down below 0ºC (up to -10 ºC) [17,45,46].*

**Line 133: Is “yoks” supposed to be “yaks”?**

***Response:*** *Corrected*

**Lines 144-151: This was all stated in introduction already, so is not needed here.**

***Response:*** *Corrected*

**Lines 156-160: Put this description of the valleys into the previous section (Study Area).**

***Response:*** *Corrected and added to study area Please see section M&M page 7 line 133-136*

**Lines 161-162: Put this sentence into the next section (Warming and Grazing Experiments)**

***Response:* Needful done** *Added to the relevant section. see line 162*

**Lines 167-170: The RFC description should be included in the “statistical analyses” section instead.**

***Response:* Done** *Added to statistical analysis section please see section M&M page 11 line 229-231*

**Lines 171: What does “we involved plant material” mean?**

**Response:** *The specific question asked were about native plants and their medical and cultural properties. The sentence is now restructured, please see line 148-149*

**Lines 182-183: What does “which lies in alpine meadow of the vegetation zones” mean?**

**Response:** *There are four vegetation zones in which KNP is divided already described in introduction section. The study sites are in the alpine meadows of those zones Please see section introduction page 6 line 125-134.*

**Line 184: Since there was only 1 replicate for each elevation, the entire experiment is an RBD, but not each site.**

***Response:*** *Corrected, please see line 164-169*

**Lines 186-188: Were the data collected outside of this fenced area used for any analyses? I don’t see it used.**

**Response:** *we did not use the data outside of fenced area but the plots in surrounding were considered as reference to mimic the herbivory level on plant species for clipping treatment.*

**Line 191: Where was this reported earlier? Or do you mean it was reported by another prior study?**

***Response:*** *Yes, prior study the reference is given, line 176*

**Line 197: How can a hexagonal shape have the dimensions of 1.5 x 1.5 m or 1 x 1 m? This reporting style is reserved for squares/rectangles. Do each of the six sides have that length? If so, that needs to be stated.**

***Response:*** *The area covered by OTCs was inside the 2.5m^2^ subplot. The area it covered was 1.5m^2^and 1m^2^ quadrat was used to sample the species biomass and cover inside OTC that corresponded from the open top of chamber. Please refer to Figure 1 line 202*

**Line 198: Since your fences prevented grazing, they should be called “exclosures” instead of enclosures throughout the**

**paper.**

***Response:*** *Needful done*

**Lines 212-214: This is confusing.**

*The experiment consisted of two treatments, one 2.5m^2^ subplot inside exclosure subjected to clipping treatment, one similar dimensioned plot outside the fenced area as reference to estimate the herbivory level on plant species in order to mimic the grazing. Line 190*

**Line 214: Define the abbreviation “W x CL” the first time it’s used.**

***Response:*** *Needful done. please see* *line 193*

**Line 216: Simulate “grazing” (instead of “growth”)?**

**Response:** *Corrected*

**Lines 218-219: What is described here? I don’t understand the 2 grazing comparisons. The analyses only discuss the manually clipped plots.**

**Response:** *The grazing plots outside the fenced area is used as reference to check the level of herbivory outside and then employing it to clipping treatment. the intention is not the comparison.*

**Line 220-221: This is confusing.**

**Response: Corrected as follows** *Please see section M&M page 10 line 197-201*

*At the start of experiment, the grazing level of herbivores was estimated and clipped inside the exclosure in clipping treatment, then each year plant growth was recorded from that clipped point. In the clipped plots the aboveground part of all selected plant species were clipped to about 2–4 cm on an average above the entire plot. There was no disruption in the unclipped plots.*

**Line 224-225: Remove this sentence.**

**Response:** *Needful done*

**Line 132: How large were the quadrats and the quadrat grids?**

***Response:*** *1m* x *1m quadrat plot with 10cm quadrat grids. Please see line 217-219*

**Line 234: How were the “selected plant species” determined?**

**Response:** *The plant species were selected based on their medicinal and cultural potential mentioned by informants, citation frequency, occurrence in alpine meadows and also the palatability as described by local inhabitants Please see section M&M page 10 line 215-217*

**Lines 236-241: Put all the species into a table and reference it instead.**

**Response***: Yes, it would be wonderful to add them into a table with reference, but that would be a repetition as these are already in the tabulated form in Table 1 and S3Table. The reference to Table 1 is given*

**Lines 243-244: What does “vertical projections of vegetation” mean?**

**Response:** *That is how cover is defined, as to estimate the area covered by a plant*

*Daubenmire, R. 1959. A canopy-coverage method of vegetational analysis. Northwest Sci. 33:43-64*

**Line 248: How large were the clipping areas? Were the same areas clipped multiple years in a row?**

*The clipping treatment area was 2.5* x *2.5m at each site, Yes, the same area was clipped in a row*

**Line 250-252: I’m unclear about what you used to calculate % increase/decrease for the data. If the values were averaged over all 3 years, how was % increase/decrease calculated as the “difference between control and last year data**

**points”?**

**Response:** *yes, it was calculated as difference between control and last year data. % increase= last year cover/cover in control x100. If the number is positive, then its increase and if negative, it’s percent decrease. Please see line 224-226*

**Lines 155-156: This sentence not needed here.**

**Response:** *It is removed*

**Line 290: Missing % for Asteraceae**

*Added*

**Lines 291-293: Unclear**

*According to the reference given, if a plant species is mentioned or cited for a typical trait by more than three informants it is considered as reliable medicinal property [62], please see 15 ;line 270*

**Lines 301-302: This sentence not needed.**

**Response:** *removed*

**Lines 306-308: Save for discussion section**

***Response:*** *Added in discussion section, line 395.*

**Lines 308-309: This is confusing.**

**Response**: *The sentence is removed as it has already explained at the start of paragraph*

**Lines 313-315: This sentence not needed here.**

**Response:** *Needful done*

**Line 321: What does “across all three growing seasons” mean in this context? Average over 3 years? The value at the end of year 3? Each year assessed independently (then year would need to be included in the statistical model)?**

***Response:*** *this section is removed from the manuscript and only mixed effect model results are shown. Please see results*

**Line 425: Plant height was not statistically analyzed or discussed in results section, so should not be mentioned here.**

***Response:*** *It is removed*

**Lines 425-428: Unclear**

**Response**: *It is clarified now.* *The increase in plant species abundance and cover was observed along the elevation gradient but this effect was more profound for specific species which increased their vegetation cover and biomass in warmer plots, but reduced biodiversity. Please see page 21 line 403*

**Line 456: State author’s names (instead of reference number) if specifically referring to a study.**

**Response: Needful done.** *Please see line 439*

**Lines 490-494: Unclear**

***Response:*** *It is clarified. Please see page 25 line 478-480*

*The intensity of positive response was higher in the zone of warming as higher percentage cover and biomass (Fig 4), thus predicted the vulnerability of these species towards herbivores as more availability more grazing [78]*

**Lines 496-497: How does the data support this assumption?**

***Response:*** *Our data supports the natural selection as some species has higher biomass and cover in warming plot than others. please see page 25 line 486-490*

*This supports the assumption of natural selection where strong competitor species surpass weak competitors thus lead to loss of biodiversity[34,53]. These experimental evidences were also in line with the information gathered from the local informants, mentioning increased forage availability of these species over the period of climate warming.*

**Lines 500-505: I like the biodiversity discussion. Expand on this more.**

**Response:** *Needful done. please see page 25 line 491-513*

**Figures:**

**Figure 1: The figure legend needs to include much more detail – it needs to fully describe each component of the plot design. For example, why are there control plots in the warming and warming x clipping larger plots? What do the letters on the OTC mean? Unclear what the 1 m and 1.5 m arrow are pointing to.**

**Response:** *Figure 1: the details are added in the caption to best illustrate the experimental design and explain the sampling sites for species composition and biomass estimation. The schematic diagram has been improved with more clarification for plots and subplots. Please see Figure 1 page 10 line 202*

**Figure 2A: First, these should be bar charts or box and whisker plots instead of line graphs. Line graphs are usually used to represent changes through time. What do the stars signify? Panel A needs a better y-axis label (no shortened words, underscores).**

**Response:** *It is* *suggested to change it into box and whiskers plot with suggestion that line graphs represent changes over time. It is humbly mentioned here that this statement supports to our study data too, we also studied climate warming impacts over a period of 3-years. Thus, we request to consider the Fig 2 in line graphical form. For Panel A, needful done as suggested.*

*Figure 2 as box and whisker plot is also submitted as a separate file and with the rebuttal letter just for your kind perusal.*

*Linear mixed effect model representation of significant effects of each treatment (Fixed effect) on percentage cover and biomass. a) As compared to control percentage cover was higher in warming plots(p<0.01) but Warm and clip treatment significantly had more cover of species but less than warming plots suggesting that clipping affects the overall percent cover. b) greater biomass of all species in warming treatment as compared to control and other treatments, suggesting that under passive heating plants tend to increase their vegetative growth. The stars represent the significant level. The significant codes are 0 ‘***’ 0.001 ‘**’ 0.01 ‘*’ 0.05 ‘.’ 0.1 ‘ ’ 1*

**Legend line 348-349: Is there statistical evidence for this claim?**

***Response****: This line is improved as above in the legend of figure 2*

**Line 349-351: This is not needed here.**

**Response:** *removed*

**Figure 2B: First, why is this labelled “2B”? It appears to be an entirely separate figure, so should be “Fig 3.” What do the stars and dots represent? The axis labels should be written without underscores. Also, why does it appear that most of the control plots increased? How is it that none happened to decrease over the 3-yr period?**

**Response:** *This figure is updated as Fig 3. Here is the improved complete legend of it*

**Fig 3**: S*pecies-specific responses towards each treatment.* ***a)*** *data showing the percent increase in cover of each species in control, warmed, and clipped plots* ***b)*** *in above ground biomass of each species in response to warming, clipping, and warming* x *clipping compared to control, warming has overall positive effect on percentage cover and biomass of species while clipping has negative effect. The * shows a significant change in particular species for the specific treatment while the dots are outliners.*

**Figure 3: You explain in your review response to what the “beans” represent, but this description needs to be included in the figure legend. It would be helpful to label each panel as “warming” or “clipping” depending on what it shows. It might also be nice to identify (maybe by color) which species are the most culturally important- it ties this data to the survey responses that way. Again, there are still no letters indicating statistical significance (as stated in the legend). The stars show statistical significance of each bar compared to what- each other.**

***Response:*** *As this Figure was in repetition with figure 3 so it is removed now*

**Figure 4: What do the stars mean?**

*The significant positive effect of warming on species percent cover and biomass in absence of grazing*

**Figure S1: Improve the y-axis label**

***Response:* *Needful done.*** *Improved and full form of the y-axis is added.*

**Figure S2: Similar to figure 2A, these should all be bar chars or box plots. Improve axis labels. For the right panels, reorder species to group by cultural importance or functional group. Much more detailed legend needed.**

**Response:** *As a supplementary figure, this has been kept as it is, because it is not the main result but as a supporting information, and the information given in it has already been explained in figure 2 and figure 3, but for sites as a fixed factor this can be important information. The order of treatment has been changed.*

**Figure S3: Omit or re-structure this figure- it’s unreadable.**

***Response:*** *Needful done.*

**Figure S4: What do the different colored dots represent? It’s hard to tell which labels go with which points. I’m not familiar with these types of plots, but this is confusing to me. Maybe put more description in the legend?**

***Response:* NMDS (Non-metric multi-dimensional plots) used to determine the relative difference between the species in a community over the period of a particular time**

*Non-metric multidimensional scaling (NMDS) plot of plant species biomass-with 95% confidence intervals using Bray and Curtis dissimilarity index over a 3–years experimental period along the elevation gradient of (3590–4696m). The relative abundance of plant species biomass inside the OTCs on each elevation site was estimated for all study years and shown by paths of mean values in Non-metric multi-dimensional scaling (NMDS) using Bray and Curtis dissimilarity index in R The plot shows no significant difference between the relative abundance of there is no significant change in the relative abundance of species inside both OTCs.*

*OTC = Open top chamber, OTC1(warmed), OTC2, (warm*clip). Site 1 4,696m, Site 2= 4,059m Site 3= 4022m Site4=3,990m Site 5= 3,590mDifferent colour represents each study year, red, 2016, green, 2017, blue 2018. Please see Figure S3*

**Table S2: Thanks for editing this table. One suggestion – make separate columns for each elevation, and then put an X in**

**each column where a species is present at that elevation. Right now, you can’t tell which species are present at which**

**elevation.**

***Response:*** *corrected please see S2 table*

**Table S3 & S5: Include the full ANOVA tables/statistical output. More than just the p-values should be included.**

***Response****: As the analysis were repeated with the linear effect model so we removed the un-necessary results and figures and only kept the results of mixed effect model keeping the effect of site and species as random. Thus S3_tables are removed, only the species-specific responses are added and the values are represented as mean ± SE and more information as added to Table 2*

**Data points: Need metadata- full description of what each dataset it and the meaning of each abbreviation.**

***Response:*** *The data points excel sheet is now edited with full form and description of abbreviations*
