# Supplementary material for: The response of culturally important plants to experimental warming and clipping in Pakistan Himalayas
Source: PLoS One. 2021 May 6;16(5):e0237893. doi: 10.1371/journal.pone.0237893 (PMC8101745; doi:10.1371/journal.pone.0237893)
Supplement: Attachment — Submitted filename: Response to reviewers_3.docx [file pone.0237893.s011.docx]

**Authors Response to Reviewers**

**Journal Requirements:**

**Please review your reference list to ensure that it is complete and correct. If you have cited papers that have been retracted, please include the rationale for doing so in the manuscript text or remove these references and replace them with relevant current references. Any changes to the reference list should be mentioned in the rebuttal letter that accompanies your revised manuscript. If you need to cite a retracted article, indicate the article’s retracted status in the References list and include a citation and full reference for the retraction notice.
*Response:*** *Reference list is updated*

**Additional Editor Comments (if provided):**

**Dear Authors,
There are still some minor issues in statistical testing and language. Please address reviewer is all comment adequately and resubmit.**

**Reviewer #1: General comment**

**The manuscript has hugely improved and is much easier to read and understand. There are still some issues of confusion with the statistics, including the "Factor x species" model on line 240, use of t-test to assess the significances of the treatments, and understanding/reporting/interpretation of the interactive effect.**

***Response****:* ***Factor* x *species****:* *The confusion in statistics is removed. Following details are mentioned. Please see line 243-254 in M&M.*

*Here the factors are the treatments, (warming, clipping and warming* x *clipping). The model formulation was to check the individual response or species-specific response to each treatment. The summary statistics are shown in S4Table. (Please see supplementary information S4Table)*

*To estimate the fixed effects of the treatments, we used linear mixed effect models using lme4 package (Bates, Mächler, Bolker, & Walker, 2015) in R (The R Foundation, 2018). The model summary provides the estimation, standard error, and t values of each of the factors as shown in Table 2. It is shown that inferences for the fixed-effects parameters in linear mixed models are based on t-value distributions with suitably adjusted degrees of freedom. In mixed effect models, the main rationale is that while p-values can be misleading because of unclear null distribution, t values can still be useful as standardized parameters. The t-test used to evaluate the significance of overall treatments, the variance between the treatments and the response of each species to the specific treatment.*

**Reviewer comment: Interactive effects of warming and clipping treatments in OTC x clipping plot.**

***Response***:

*The overall interactive effects of both treatments have been described with more clarification. The interpretation is improved, and cross checked with the model tables and figures. Please see line 368-371, Table 2 and Figure 3. Details are as follows.*

*The mixed effect model showed that the interactive effects of warming and clipping were comparatively weaker than the main effects, both on aboveground biomass and percentage cover. Whereas clipping has a consistent negative effect in main effect plots, it decreased the aboveground biomass of the species in interactive plots too. Thus, concluded that the negative clipping treatment effect decreased the cover and percentage also warming effect was not significant in these interactive plots.*

*Also Please see section Discussion line 483*

*To better place our results in this context we explained the interactive effects as stronger clipping effect being an important finding than warming. The relative effects of warming and clipping on vegetation growth are not fully understood. The warming positive effect was not consistent in the interaction plot explaining the plants' sensitivity to combined stress.*

**Reviewer comment: Furthermore, the authors present results for the different sites for which the description of the analysis and model output tables are missing. Specific comments marked with * are particularly important.**

***Response****: The results are not described for separate sites, but we consider effect of site as random. The model output table, where each factor (treatment, site, and species) considered as fixed effect was removed as suggested by previous reviewer. It has been now added to the supplementary file as S3Table b. Please see supplementary information S3Table b.*

**Reviewer comment There is still some language that is confusing or unclear in wording, especially in the results and discussion. Also, please check for upper- and lower-case letters throughout your figures and tables and chose a consistent style. Consistency is also needed concerning the terminology of the clipping/grazing treatment and aboveground biomass.**

***Response:*** *Needful done. The terminology of clipping is used throughout the manuscript for consistency and careful revision of the manuscript is done for upper- and lower-case letters in figures and tables.*

**Reviewer comment: The authors state that "Yes - all data are fully available without restriction" and "All relevant data are within the manuscript and its Supporting Information files." but we see NO raw data in the supporting information file, just the supplemental tables with output. We hope this is an accidental error, but it needs to be corrected to meet the journal data requirements.**

***Response:*** *Needful done. Data files of all relevant underlying data point is available as supplementary excel file.*

**Running title and abstract
Reviewer Comment: Line 13: Lowercase “H” in herbivory**

***Response****: Needful done Please see line 14*

**Reviewer Comment**: **Line 22-23**: **This sentence may be easier to understand and read if you wrote: “****Experimental warming increased biomass and percent cover throughout the experiment”. It is not necessary to report the p-value or that you used sites as a random factor. The abstract should highlight your main findings in a concise form, so avoid any unnecessary detail.**

***Response:*** *Needful done. P value are removed. Sentence is added as above. Please see line 22 in Abstract section.*

**Reviewer Comment:**  **Line 27: Unclear what “due to its abundance at the site” means.**

***Response****: Poa alpina was one of the dominant species at all experimental sites but we pooled the data of percentage cover for 3 years and represented the overall increase/decrease. The words “due to its abundance at the site” have been removed. Please see line 26 abstract section*

**Reviewer Comment: Line 28: Had instead of “has”.**

***Response:*** *Needful done. Please see line 26*

**Reviewer Comment**: **Line 29: For consistency, it would be good to give the same names to your treatments. Clipping and simulated grazing are the same, right? Can you change this?**

***Response:*** *Thank you for the suggestion. Clipping is now the consistent term used throughout the manuscript.*

**Reviewer Comment**: **Line 31: Stronger effects, what does this mean? Please, report whether this refers to an increase or decrease.**

**Response:** Needful done. *The stronger effects of clipping refer to the decrease of percentage cover and biomass. Please see line 29-30*

**Introduction
Reviewer’s comment: Line 58-59: Sentence unclear, maybe the “to” needs to be replaced with “for”?**

***Response*:** *Needful done. Please see line 56 in introduction section* **Reviewer’s comment**: **Line 60: Delete the “s” at the end of “causes”.**

***Response:*** *Needful done*

**Reviewer’s comment**: **Line 83: underrepresented?**

***Response:*** *Needful done. Changed from underrepresented to marginalized. Please see line 81*

**Reviewer’s comment**: **Line 84-85: Sentence unclear.**

***Response:*** *The sentence has improved as.* ***“****The incorporation of indigenous knowledge of medicinal plants with the current biological conservational practices is necessary to assess the future face of biodiversity and associated threats”*. *Please see line 82-84*

**Reviewer’s comment**: **Line 101: This is the first time your mention KNP, please provide an explanation of the abbreviation.**

***Response****: Needful done. Please see line 100*

**Methods:**
**Reviewer’s comment**: **Line 117: No brackets needed when you mention the altitude.**

***Response:*** *Needful done. Please see line 117* **Reviewer’s comment**: **Line 126: Parking area??**

***Response:*** *corrected as Park area. Please see line 126*

**Reviewer’s comment**: **Line 132: Comma missing between lanceolata and Saxifraga.
*Response:*** *Needful done. Please see line 132*

**Reviewer’s comment**: **Line 139: I think you mean individuals instead of species here.**

***Response:*** *Needful done. Please see line 139*
**Reviewer’s comment**: **Line 190: Here you write that your clipping plot inside the fence is 2.5 m2, but from Figure 1 it looks like it is 1m2. Please clarify this.**

***Response***: *The subplot designated to the clipping treatment is 2.5m x 2.5m. By 1m* x *1m dimensions means that we clipped the biomass from 1m* x *1m area by quadrat method. Please see line 211 in data sampling section of material and methods*

**Reviewer comment:** **Line 193: Could you add the clipped area inside the OTC to Figure 1**?

***Response***: Needful done. The area where species composition is mentioned was clipped and shown in Figure 1. *Please see the Figure 1*
**Reviewer comment:** **Line 209: Lowercase “a” in “all”**.

***Response***: *Needful done. Please see line 209*

**Reviewer’s comment:** **Line 212: As pointed out in an earlier review, this reference seems not appropriate for plant metrics, but focusses on ground dwelling insects in riverbeds. You could cite for example Shaver and Chapin 1991: Production: biomass relationships and element cycling in contrasting arctic vegetation types, Ecological Monographs.**

***Response****: Needful done. The updated reference is added as follows and updated in text too as[56]: Please see reference line 717 and line 212 in methods*

*Sanaei A, Ali A, Ahmadaali K, Jahantab E. Generalized and species-specific prediction models for aboveground biomass in semi-steppe rangelands. J Plant Ecol. 2019;12: 428–437. doi:10.1093/jpe/rty037*

**Reviewer’s comment: Line 223-224: What do you mean with “We clipped the vegetation from OTC”? This sentence needs clarification.**

***Response:*** *Needful done. We measured aboveground biomass of each individual species in each treatment and OTC too by clipping the whole species using quadrat method, sort it out separately and weighed in laboratory by air drying at 37°C for 72 hours. Please see line 224*

**Reviewer’s comment: * Line 240: The model formulation for this second, species-specific model is not clear. What is "Factor" in this model? I think what you are intending to write is Factor * species + (1|site)?**

****Response:*** *Factor in the model is the treatment [warming, clipping and warm*clip (interactive treatment)]. We described the species-specific response to each treatment. (Please see S4Table). The model formulation is updated as follows.* *Perc cover ~Factor*species: (1|site). Please see line 243*

**Reviewer’s comment: Line 241 -244: Sentence unclear.**

***Response: The sentence is re-structured as”*** *The high ethnomedicinal value (Table 1) and the response to the treatments illustrated by mixed effect models, the vulnerability and susceptibility of important plant species was determined. These species included Artemisia rupetris, Poa alpina, Primula macrophylla, Potentilla hololeuca and, Astragulus penduncularis. The combined effect of treatments on these species was illustrated as susceptibility zones to show the positive and negative effects of both the treatments and analyzed the occurrence of constitutive species.* *Please see line 256-263*

**Reviewer’s comment**: ***Line 244: “polygons- based susceptibility zones” unclear. If this is an established method, you should provide references**.

***Response***: *It is graphical illustration of the results obtained by linear mixed effect models (S4 Table). From the results the significance of interactive treatment effect was shown in figure 4 as susceptibility of the species to the combined effect of each treatment. The clipping and warming treatment effect was drawn as four susceptibility zones whose combined effect would increase or decrease the cover and biomass of highly important medicinal plant species. Please see line 256-263*

**Reviewer’s comment**: * **Line 246: You should add here which kind if test you used to assess the significances of the treatments, and what function or package you used for that test.**

***Response****:* *Needful done. The given information is added to the text.* *The significances of treatments were assessed by linear mixed effect models for random (site, species) and fixed (treatments) effects using lme4 package, function lme(). The plot was made by ggplot2, function geom_polygon, package tidyverse. Please see above response also line 261-263*

**Reviewer’s comment**: **Line 250: Point missing between “groups” and “the”**.
***Response***: *Needful done. Please see line 255*
**Results**
**Reviewer’s comment: Line 267: Percentage for Asteraceae is missing.**

***Response****: Needful done. Please see line 284*

**Reviewer’s comment:** Line 295: **You state that clipping is not significant, but in Figure 2a there are three asterisks to indicate high significance. Same for the interaction which is not significant according to Table2. Also based on Table 2 there should be two asterisks for the interaction the Figure 2b and only one for the clipping treatment.**

***Response:*** *Needful done. The text and figure are updated according to the significance mentioned in Table 2. Please see Figure 2-part b and text line 310-316. The further details are given below:*

*Contrastingly clipping exerted significant negative effect on biomass and non-significant negative effect on percentage cover. Plant species decreased their aboveground biomass in clipping plots while there was a significant increase in biomass in warming plots. This positive effect was overridden by the clipping effect in interaction plots where the combined impact of both warming and clipping was found to be significantly negative on aboveground biomass (p<0.05) (Table 2).*

**Reviewer’s comment**: **Line 307: Why two models? What you show in the table can be derived from one model which contains both the main effects and the interaction.**

***Response****: The sentence is structured as follows:*

*Mixed effect model summary showing estimate, standard error, and t statistics is described in Table 2 explaining the significant fixed effects of experimental warming, clipping and the interaction treatment (Warm*Clip) on plant species. Please see line 320-322*

**Reviewer’s comment**: **Line 314 – 316: Species is missing an “s”, the sentence is unclear.**

***Response:*** *Needful done.* *Species specific response to each treatment while keeping the effect of the site as random showed that elevated warming has significantly increased the percentage cover and aboveground biomass as shown in Figure 2, S4Table. Please see line 325-327*

**Reviewer’s comment: Line 318 -320: This sentence could be moved to the results but is not needed to describe the figure. You already do that in a) and b).**

***Response:*** *This sentence is removed. Please see line 329-330*

**Reviewer’s comment: Line 319: Write the full name of the treatment, not just “warm” and “clip”. It is always good to be consistent in the use of treatment names/terminology!**

***Response****: Needful done. This sentence is removed, please see the comment above. Moreover, the whole manuscript is revised for the warming and clipping term consistency.*

**Line 319 – 320: If clipping had a consistent negative effect you would see this as a main effect. Here you need to think about why the combination of warming and clipping decreases percentage cover.**

***Response:*** *Our study reported consistent positive warming and negative clipping effects while in the interaction treatment plots clipping effect dampened the positive effects of warming because clipping usually counteracted the warming effect. Clipping has stronger effects than the warming also the alpine vegetation responses to combined stress suggested that clipping(grazing) is responsible driver for the vegetative productivity in the region. Warming enhanced the percentage cover but in this plot plant species are under the pressure of two main treatments, so consistent negative impact if clipping here has outcompete the positive effects on vegetation. It suggested it is difficult to predict the combined effects of change from single factor studies. This is later explained and discussed in results and discussion session. Please see line 482.*

**Reviewer’s comment: Line 322: See the above comment. In general, you do not need to add interpretations of the results to the figure caption, reserve this for the discussion.**

**Response:** *Needful done. Please see line 331*

**Reviewer’s comment: * Line 326: You did not mention in your statistical analysis that you assessed differences in percentage cover and biomass between the different sites. Please add this information. Furthermore, you should also provide a table with the model output. Referring to the figure only is not sufficient because the reader cannot judge based on a figure whether something is significant or not.**

***Response:*** *Needful done.* *In statistical analysis, factor effect plot analysis is mentioned. Please see line 228.*

*The factor effect plots of treatment site and species were added with the model output statistical summary in early draft. But because we took site as the random effect, reviewer has asked to remove these table. The individual effect of each factor was determined (S2Figure) to know the significance of each treatment on cover and biomass keeping each factor as fixed in mixed effect model. The model output is added as S3Table b, c&d where the significance of each factor was determined on percent cover and biomass. Please see supplementary information S3Table.*

**Reviewer’s comment: Line 330 – 333: Sentence unclear and confusing.**

***Response:*** *We made the factor effect plots for each variable (site, species, and treatment). The main effect was of the treatments and site and species were taken as random effects. But we individually determined the effect of each factor as well (S2Figure)**. The response variability was high among the highest elevation site (M1) and the lowest (M5) suggesting that site may be a determining factor for difference in response magnitude of the species.* *Changes in the species composition along the elevational gradient as random effect can be described as predictor about the variance in response between different sites. Please see lines 337-340*

**Reviewer’s comment: Line 336 – 337: Higher biomass is a result of warming, but warming is not determined by higher biomass. Please, rephrase this.**

***Response:*** *Needful done.* *The overall differences in response magnitude of all species' percentage cover and biomass can be seen in species effect plot where some plant species as Poa alpina (PA), Astragulus penduncularis (AS), Artemisia rupestris (AR), and Potentilla hololuca (PT) had higher percent cover and biomass in comparison to others (S3Table a, S2Fig). Please see line 344-348*

**Reviewers comment: Line 337: Figure S2 species-specific plots, are these plots in response to warming or in response to clipping or both? What does this figure add in comparison to figure 3?**

***Response****: The effect of each factor was determined before selecting the fixed and rando factors in developing mixed effect models. Figure S2 explains the overall factor effect considering the species as fixed factors while the Figure 3 explains the species-specific response and effect of individual treatment on each species. It was added as main figure before, but it is moved to supplementary information as per reviewer’s suggestion*

**Reviewer’s comment**: **Line 343: Comma missing between ruprestis and Poa.**

***Response:*** *Needful done. Please see line 353*
**Reviewer’s comment: Line 353: Using “suggest” here seems not fitting, rather use “show”.**

***Response****: Needful done. Please see line 369*
**Reviewer’s comment**: **Line 356 – 357: This seems to be the same information than in the beginning of the paragraph just in other words. Consider deleting this?**

***Response***: *Needful done. Please see line 371-374*

**Reviewer’s comment**: **Line 358-359: Instead of “relationship” it would be better to use “effect” here. Also, not sure what you want to say here**.

***Response:*** *Needful done. The whole sentence is re-structured for clarification of interactive experiment result. Please see line 369-375*

*Mixed effect model showed that the interactive effects of both warming and clipping were comparatively weaker than the main effects, both on aboveground biomass and percentage cover. Whereas clipping has a consistent negative effect in main effect plots, it decreased the aboveground biomass of the species in interactive plots too. Our finding showed that response magnitude of percentage cover and aboveground biomass to clipping was stronger than the experimental warming in interaction (W* x *CL) plots (Figure 2, Table 2). It has decreased the aboveground biomass of species significantly (<0.01) as shown in Figure 2 and Table 2.*

**Reviewer’s comment: Line 361: “though” may be the wrong word here.**

***Response:*** *Needful done. Please see comment above*
**Reviewer’s comment:** **Line 362: Something is missing in this sentence, most ….?**

***Response:*** *The whole paragraph is re-structured. Please see response to comment line 358-359*

**Reviewer’s comment:** Line 365: There is no S4 figure in the supplement.

***Response:*** *The S4 Figure is now S3 Figure. Please see supplementary figures S3Figure*
**Reviewer’s comment: Line 370: Do you mean increase or decrease? In the previous part of the sentence, you write about a negative effect of clipping, so I assume it should be decrease. Again, it is confusing that you write clipping was not statistically significant, but Fig.2 shows significant asterisks for the clipping treatment.**

***Response***: *The overall effect of treatment and species-specific response is given in two figures figure 2 and figure 3. It is corrected as our finding showed that response magnitude of percentage cover and aboveground biomass to clipping was stronger than the experimental warming in interaction (W* x *CL) plots (Figure 2, Table 2). It has decreased the aboveground biomass of species significantly (<0.01) as shown in Figure 2 and Table 2. Please see lines 372-375*

**Reviewer’s comment**: **Line 381ff: You describe panel a) but not panel b) in your figure. The meaning of “…than clipping” is unclear, could be removed?**

**Response:** *Needful done. Please see lines 390-391*

**Discussion
Reviewer’s comment: Line 392: In the results you say that clipping has NO significant effect!**

**Response**: *It is corrected in the results section “clipping effect” too.*

*The clipping effect on biomass was significantly negative (Table 2) it means that in main clipping plot and interaction plot the biomass was reduced. Please see line 460-462*

**Reviewers comment Line 394: Insert “increasing temperature” here, because OTCs are used to study effects of warming and not just temperature.**

**Response:** *Needful done Please see line 408*
**Reviewer comment: Line 404: enhanced**

***Response:*** *Needful done Please see line 423*
**Reviewer comment: Line 405ff: Try to focus only on the warming treatment and not suddenly list discussion points related to the clipping treatment, since you will have a separate paragraph for it later. I suggest moving all discussions relevant to clipping treatment to the appropriate paragraph.**

***Response:*** *All discussion points related to warming have been moved to experimental warming section of discussion. Please see lines 430*
**Reviewer comment** **Line 435 ff: I suggest moving all information related to warming to the previous paragraph. Otherwise, it's confusing to read about the ethnobotanical survey and then jump back to warming.**

**Response**: *Needful done. Please see comment above*

**Reviewer’s comment**: **Line 458: “… thus allowing natural selection to favor herbivores” does not sound right.**

***Response*:** *removed. Please see line 472-475*
**Reviewer’s comment**: **Line 463 – 464: The sentence starting with “therefore” is unclear.**

***Response:*** *It is rephrased as:* ***“****our results supported this assumption, where clipping treatments had significant negative effects on plant biomass and non-significant negative effects on percentage cover. The general trend was decline this decrease was highly variable among the taxa also the random effect of site can also be a predictor of variability in response[77]. Please see line 478-482*

**Line 468:** **For consistency please decide whether you want to use AGB, aboveground biomass or biomass throughout the manuscript. If you use too many terms for the same variable, it becomes very confusing.**

***Response:*** *The unified term “aboveground biomass” is used consistently throughout the manuscript and any other confusing term has removed*
**Reviewers comment: Line 477 -479: Sentence unclear.**

**Response:** *Please see line 496-501* **Line 479 – 481: The reasoning here is unclear and not convincing. Your interaction shows that the positive warming effect is strongly reduced by clipping, so in my opinion you should discuss why grazing overrides the effect of warming.**

***Response***: *Needful done*. *Please see line 496-512*

*The magnitude of positive responses of species as increased percentage cover and aboveground biomass was in the zones of only warming treatment(+W,-G), in absence of clipping (Fig 4), thus predicting the availability of these species as preferred forage if the grazing is controlled. [78]. In contrast, above ground biomass has significantly decreased in clipping and interactive plots and strongly reduced the positive impacts of warming. This outcome was most likely recognized to the following mechanisms. The relatively small effects of experimental warming than clipping in interactive plots on vegetation biomass and cover can be related to experimental warming induced soil drying. The plant species are under the stress of two climatic drivers. Thus, warming has negligible positive effects on vegetation in presence of clipping. Grazing is reported to have strong effects on C storage than warming it can significantly decrease leaf area and plant photosynthesis[78–80]. This mechanism explained the relatively large effects of clipping on vegetation biomass which overrides the warming effect in interaction plots. Again the species specific response of these taxa is shown in biomass susceptibility zone where some species as A.rupestris, and P.alpina has non-significantly increased the biomass.* *Thus, predicting future vegetation in alpine ecosystem requires the knowledge of grazing interaction with climate variables which can influence the dynamics of alpine ecosystem.*

**Reviewer comment: Line 485: Figure 2 does not show the information you are referring to. Figure S2 shows species specific responses, but it is unclear whether these are in response to the combination of warming and clipping. Please, clarify both the figure and the text!**

**Response:** *Needful done. The figure 3b is added in place of figure 2 Please see line 514.*

**Reviewer comment Line 501: What do you mean with indirect warming effects. Please explain in the text.**

**Response:** *Please see lines 530-531*. ….. *may differ in their ability to measure the indirect impacts (human and environment interaction, health risks through rising air temperatures and heat waves) of climate warming but can provide a very rapid estimation of short-term direct impacts.*
**Reviewer comment** **Line 503 – 505: Sentence unclear.**

**Response**: *Needful done*. Please see lines 532-534.*The future prediction of alpine biodiversity of culturally important species depends highly on the optimal grazing practices in the current climate warming scenario in HKKH*
**Reviewer comment** **Line 511: Point missing before “Finally”.
*Response***: *Needful done*. *See line 541*
**References
Reviewer comment**: **Reference 29 and 31 are the same.**

***Response:*** *Needful done*. *The repetitive reference is removed.* **Reviewer comment** R**eference 75 is not related to your topic and does not support the point of the sentence in which the reference is cited.**

***Response: Needful done.*** *This reference is removed.*

**Reviewer comment** **Sometimes the references are reported in different styles e.g. number 54 includes editor names, number 74 has these lines | between names. Please check that the style of the references is consistent!
*Response:*** *Needful done*
**Figures
Reviewer comment**: * **Figure 1 Has improved, but there is still a confusing sense of scale, as the areas labeled "1m" are almost as large as the areas labeled 2.5m. Also was the species composition conducted in a hexagonal area or a square? Right now it looks inconsistent between the two treatments. What about the biomass extraction area, which is a weird shape? Where was the biomass extraction for the control and clipping-only plots?**

**Response:** *The biomass extraction was done using random quadrat method inside the control and clipping plots after the percentage cover estimation. The plant saplings were marked individually after clipping to a certain height and individual biomass was calculated for each species. Form OTC this destructive sampling of biomass extraction was done along the slopping sides of the OTCs.*

**Reviewer comment**: **Figure 2 Please, put the correct amount of * to each treatment. For now, the model output, the text and the figure are not consistent regarding the significance of the treatments.**

***Response***: *Needful done*

**Reviewer comment: Figure S2 The first graphs (a and b) are the same as Fig.2. Consider to delete these, because they take unnecessary space. Furthermore, it is unclear in the species-specific graphs whether these show responses to only one of the treatments or if the data are averages across all treatments.**

***Response****: It is worth to mention that this was main figure in the first draft of the paper. But the reviewers suggested us to put this figure into supplementary information. By this S2Figure, we intend to show the factor effect on biomass and percentage cover considering each variable as fixed effect and according to previous revision Figure S2 and Figure 2 describe our results in a best way. Though it is in supplementary information, we request to include this information in our manuscript as supplementary information.*

**Reviewer comment** **Figure S3 This does not have a clear message. Consider deleting it.**

***Response***: *The relative abundance of each plant species biomass in the warming and warm*clip treatment was estimated through Non-metric multidimensional scaling (NMDS) plot with 95% confidence intervals for over a 3–years experimental period along the elevation gradient of (3590–4696m). The relative abundance of plant species biomass inside the OTCs on each elevation site was estimated for all study years and shown by paths of mean values in Non-metric multi-dimensional scaling (NMDS) using Bray and Curtis dissimilarity index in R. The plot shows no significant difference between the relative abundance as there is no significant change in the relative abundance of species inside both OTCs.*

**Reviewer comment Figure S4 Is missing but referred to in the text.**

***Response:*** *Needful done. The number of the figures are updated*

**Tables**
**Reviewer comment:** **Table 1 Good but pay attention to consistent use of upper- and lower-case letters in the beginning of each word.**

***Response:*** *Needful done*
**Reviewer comment: Table S2 Good, though in the caption “specie” is supposed to be “species”.
*Response:*** *Corrected*

**Reviewer comment: Table S4 Please see comment to line 240.**

***Response:*** *Factor in the model is the treatment [warming, clipping and warm*clip (interactive treatment)]. We described the species-specific response to each treatment. (Please see S4Table). The model formulation is updated as follows.* *Perc cover ~Factor*species: (1|site)*
